# Supplementary material for: Assessing Ebola virus circulation in the Tshuapa province (Democratic Republic of the Congo): A One Health investigation of wildlife and human interactions
Source: PLoS Pathog. 2025 Nov 26;21(11):e1013628. doi: 10.1371/journal.ppat.1013628 (PMC12680337; doi:10.1371/journal.ppat.1013628)
Supplement: S1 Supporting Information — (DOCX) [file ppat.1013628.s011.docx]

**S1 Supporting Information. Context, methods and results.**

**Assessing Ebola virus circulation in the Tshuapa province (Democratic Republic of the Congo): A One Health investigation of wildlife and human interactions**

Rianne van Vredendaal^1¶*^, Léa Joffrin^1¶^, Antea Paviotti^2,3^, Claude Mande^4,5^, Solange Milolo^6^, Nicolas Laurent^7^, Léa Fourchault^1,7^, Douglas Adroabadrio^4^, Pascal Baelo^4^, Steve Ngoy^4^, Papy Ansobi^8^, Casimir Nebesse^4,5^, Martine Peeters^9^, Ahidjo Ayouba^9^, Maeliss Champagne^9^, Julie Bouillin^9^, Jana Tĕšíková^1^, Natalie Van Houtte^1^, Sophie Gryseels^1,7^, Maha Salloum^2,3^, Freddy Bikioli^6^, Séverine Thys^10^, Jimmy Mpato^11^, Ruben Ilonga^12^, Henri Kimina^13^, Ynke Larivière^2,3^, Gwen Lemey^2,3^, Pierre Van Damme^2^, Jean-Pierre Van Geertruyden^3^, Hypolite Muhindo-Mavoko^6^, Patrick Mitashi^6^, Herwig Leirs^1^, Erik Verheyen^1,7^, Guy-Crispin Gembu^4,5^, Joachim Mariën^1,14^

^1^Evolutionary Ecology Group, Department of Biology, University of Antwerp, Antwerp, Belgium

^2^Centre for the Evaluation of Vaccination, Vaccine and Infectious Disease Institute, University of Antwerp, Antwerp, Belgium

^3^Global Health Institute, Department of Family Medicine and Population Health, University of Antwerp, Wilrijk Belgium

^4^Centre for Biodiversity Monitoring, University of Kisangani, Kisangani, Democratic Republic of Congo

^5^Department of Ecology and Wildlife Management, University of Kisangani, Kisangani, Democratic Republic of Congo

^6^Tropical Medicine Department, University of Kinshasa, Kinshasa, Democratic Republic of Congo

^7^Vertebrate group, Directorate Taxonomy and Phylogeny, Royal Belgian Institute of Natural Sciences, Brussels, Belgium

^8^Ecology and Control of Infectious Diseases Unit, Basic Sciences Department, University of Kinshasa, Kinshasa, Democratic Republic of Congo

^9^TransVIHMI, University of Montpellier, Institute for Research and Sustainable Development (IRD), INSERM, Montpellier, France

^10^ASTRE, French Agricultural Research Centre for International Development (CIRAD), Montpellier, France

^11^Boende General Hospital of Reference, Boende, Democratic Republic of Congo

^12^Central Office of Boende Health District, Boende, Democratic Republic of Congo

^13^Anthropology Department, University of Kinshasa, Kinshasa, Democratic Republic of Congo

^14^Virus Ecology group, Department of Biomedical Sciences, Institute of Tropical Medicine, Antwerp, Belgium

*Corresponding author

Email: [rianne.vv@outlook.com](mailto:rianne.vv@outlook.com) (RVV)

^¶^These authors contributed equally to this work.

**Context**

A substantial number of animals have been captured, sampled, and screened between 1978 and 2023 in the DRC to detect the EBOV animal reservoir (S1 Table) (1–8). Interestingly, the studies conducted a few weeks after the start of an EBOV outbreak in humans did not seem to have higher detection rates.

Certain species of bats are suspected to serve as EBOV reservoirs based on biological evidence: EBOV antibodies were detected in at least eight frugivorous and two insectivorous Old World bat species (3,4,9–14), and viral RNA in three of these species (*Epomops franqueti*, *Hypsignathus monstrosus*, and *Myonycteris torquata*) (15). Additionally, certain bat species (*Mops condylurus, Chaerephon pumilus, and Epomophorus wahlbergi*) are, to our knowledge, the only mammals that can survive inoculation of EBOV and replicate the virus without showing signs of the disease (16). Faecal shedding of the virus has been detected in *E. wahlbergi*, indicating a possible transmission route (16). Experimental inoculation with EBOV has also been performed on *Rousettus aegyptiacus*, in which individuals showed antibody development but no or infrequent viral detection and no evidence of viral shedding (17,18). Additionally, *R. aegyptiacus* is the established natural reservoir of a closely related filovirus, Marburg virus (MARV) (19), and *M. condylurus* bats are the suspected reservoir of BOMV (20–23). This indicates that bats play an important role in the transmission ecology of (at least certain) filoviruses.

Also, outbreaks of MARV in humans have been linked to these risk seasons (24). Detection rates of active MARV infection are high when targeting its reservoir *R. aegyptiacus*, especially during the birthing seasons. Considering the close relatedness between MARV and EBOV, a similar circulation pattern in bat populations could be expected for EBOV.

**Methods**

***Global study area***

This study was conducted in the Boende Health District, located in the Tshuapa Province in the Democratic Republic of the Congo (DRC) (S1 Fig). The region is characterised by an equatorial climate, with average monthly temperatures ranging from 23°C to 26°C and annual rainfall between 1,400 and 2,000 mm. Two relatively dry seasons (December-February and June-August) alternate with two relatively wet seasons (March-May and September-November) ([25](https://doi.org/10.1002/2014JD022586)). This climatic pattern supports a landscape composed of closed evergreen forest interspersed with human-modified areas around villages.

Within this region, Inkanamongo, a small village of approximately 750 inhabitants (centred at 20.52223°E; -0.71834°S), served as the focal site for the animal reservoir and human serology components of our study (S1 Fig). The village is surrounded by forest and accessible only via narrow forest paths. Subsistence agriculture, including cassava and rice, is practiced in fields near and deeper into the forest. Livestock such as pigs, sheep, goats, and chickens roam freely, and most houses are built from wood or bricks with thatched roofs. The local economy is largely based on the sale of wild meat and locally brewed alcohol.

Inkanamongo was selected as a focal site for several reasons: (i) it was the epicentre of the 2014 Ebola outbreak; (ii) it lies near intact forest, serving as a key access point for local hunters; (iii) it includes forest areas modified by human activity; (iv) it is home to Ebola survivors with detectable orthoebolavirus antibodies; and (v) sociological interviews were conducted both in Inkanamongo and surrounding villages, providing a combination of historical and contemporary perspectives (S1 Fig).

The study was designed to assess plausible ecological and epidemiological factors affecting EBOV transmission during a single reproductive season among selected wild host species (mainly bats, rodents, and shrews) and domestic animals (goats and pigs). Fieldwork occurred during the May–June transition from the late rainy to early dry season, when insect abundance peaks and fruits ripen, triggering increased activity among both insectivorous and frugivorous species, particularly bats, many of which are lactating or weaning young (26,27). This transitional period aligns with the seasonal window during which Ebola virus (EBOV) outbreaks have frequently occurred in the DRC, a pattern likely influenced by ecological dynamics such as bat reproductive cycles and increased human–wildlife interactions during the late rainy to early dry season (2,28,29).

***Animal Reservoir Study***

*Sampling Details*

Small terrestrial mammals were captured using a combination of Sherman live, snap, and pitfall traps to maximise the diversity. We placed 20 pitfall traps along a transect at 5-meter intervals, alternating with Sherman or snap traps positioned 5 meters away from each side of the transect. This combination of 60 traps on 3 transects was laid out in 9 different sites around the village. The sites were in primary forest, secondary forest, and old fallow land. Traps were left in the field for 8-11 days in each site, resulting in 5040 trap nights. A total of 40 Sherman live traps and snap traps were also placed in and around the houses in the villages from May 13 to June 9. Eighty-six squirrel traps were deployed in trees in the secondary forest for twenty-seven nights. Traps were checked every morning and rebaited with a mixture of palm nut pulp, nuts, and fish, depending on availability. Bats were captured with mist nets and harp traps placed in flight corridors, next to fruit trees, and above streams in the primary and secondary forests and in young fallow land around the village. Between 3 to 6 mist nets and, at most, 2 harp traps were placed for 21 nights. Bats were rehydrated when removed from traps, after process, and before release; water and sugar were used for fruit bats, only water for insectivorous ones. All animals were preliminarily identified *in situ* to genus or species level using previously published identification keys (30–33) and pictures of each animal were taken in the field. For all animals, data regarding their sex, age, and weight were recorded, and morphometric measurements were taken.

Bats were dissected (using the same methods as for rodents and shrews) only if they were found dead, unintentionally died during the collection or sampling process, were potentially from a rare or new species and needed to be preserved as voucher specimens, or if insufficient blood could be collected without endangering their lives (particularly in the case of small-sized individuals). Opportunistic samples were taken from bats that local hunters unsolicited provided. Additionally, we took rectal, oral, nasal, and urogenital swabs in DNA/RNA Shield for 34 livestock animals and 56 carcasses of large mammals (pangolins, monkeys, etc.). For the latter, we also took tongue samples in 99% ethanol.

*Viral Screening*

Based on prior studies reporting successful detection of filoviruses (Marburg virus and EBOV) in the liver and kidney (24,34–36), we conducted extractions from these specific organs. We extracted RNA from pooled kidney and liver samples of 584 individuals with the Macherey-Nagel Nucleospin RNA kit. Approximately 20 mg of tissue was used per extraction. RNA extractions of the pooled swab samples (combined with urine and faeces if available) of 370 bats were carried out using the Qiagen QIAamp Viral RNA mini kit. Swabs, faecal, and urine samples were briefly vortexed, centrifuged (1500 x g for 15 min), and pooled per individual to extract faecal RNA using the Qiagen QIAamp viral RNA kit (Qiagen, Valencia, CA, USA) following the manufacturer's recommendations. RNA extraction of swabs taken from carcasses of 56 individuals hunted for wild meat and of 34 livestock individuals was also done using the Qiagen QIAamp Viral RNA mini kit using 70 µL of each of the three or four different swabs per individual (rectal, oral, nasal, and urogenital) and adjusted with PBS in case of smaller sample volumes. Extraction was performed according to the manufacturer’s instructions, but for wild meat no carrier RNA was added and a double elution using 2 x 40 µL RNase-free H_2_O was done, while elution was in 60 µL RNase-free H_2_O for livestock. Reverse transcription was performed on 8 µL of RNA extract using Maxima Reverse Transcriptase (Thermo Scientific). Synthesised cDNA was screened for filoviruses using two PCR assays with degenerate primers targeting a fragment of the filovirus polymerase (L) gene (S2 Table).

Amplification conditions for the first system (using GoTaq from Promega) included 94°C for 2 min, followed by 40 cycles of denaturation (94°C, 1 min), annealing (52°C, 1 min), and elongation (72°C, 1 min), and a final extension step of 72°C for 5 min. The program for the first and second rounds of the nested PCR system (also using GoTaq from Promega) was denaturing at 94°C for 2 min, followed by 35 and 30 cycles (respectively) of 94°C denaturing for 30 s, 57°C and 54°C (respectively) annealing for 30 s, 72°C extending for 40 s with final extension 72°C for 5 min. The amplification products were analysed by electrophoresis in 1.5% agarose gels stained with 0.5 μg/mL GelRed Nucleic Acid Stain in TBE (Tris/Borate/EDTA) buffer. Visualisation occurred under UV light.

*Molecular* *Species Identification*

Molecular species identification was done using the RNA extracts of samples extracted with either the Qiagen QIAamp kit or the Macherey-Nagel kit as described above. DNA was extracted from the samples specified above using the Qiagen QIAamp 96 DNA QIAcube HT kit according to manufacturer’s instructions, apart from performing sample disruption with Zymo bashing beads for 15 s on max speed on a Bertin Minilys. Next, cytochrome b (cytb) PCR was performed using primers L14723 (5’-ACCAATGACATGAAAAATCATCGTT-3’) and either H15915 (5’-TCTCCATTTCTGGTTTACAAGAC-3’) or H15149 (5’-GCCCCTCAGAATGATATTTGTCCTCA-3’). Amplification conditions (using GoTaq from Promega or Platinum Taq II from ThermoFisher Scientific) included 94°C for 5 min, followed by 40 cycles of denaturation (94°C, 30 s), annealing (52°C, 30 s) and elongation (72°C, 90 s), and a final extension step of 72°C for 10 min. Positive PCR amplicons were either purified using the ExoSAP-IT protocol, following the manufacturer’s instructions and sequenced by Macrogen Europe (Netherlands) or were purified and Sanger sequenced at Neuromics Support Facility (Antwerp, Belgium). All mitochondrial raw sequences were trimmed and assembled using Geneious Prime (Biomatters Ltd., Auckland, New Zealand). The consensus sequences were aligned with ClustalW, MAFFT or MUSCLE (as implemented in Geneious Prime) to control for gaps, translate the sequences into amino acids, map the primer pairs, and trim the consensus sequences.

*For rodent and shrew samples, molecular identification was performed using a dedicated pipeline developed by Josef Bryja et al. for high-throughput species assignment in African rodent communities. This pipeline integrates alignment and taxonomic assignment steps tailored to the specific challenges of rodent and shrew barcoding in Africa. Additional information about the pipeline is available upon request.*

*Some individuals could not be identified at species level using the cytb gene and blast % identity or phylogenetic reconstruction, either because the sequence quality was not good enough or there was no reference sequence available. Cytochrome c oxidase 1 was used to identify 1 out of 10* Cercopithecus ascanius. *Other individuals were identified based on morphological characteristics. This concerned 1 out of 14* Cricetomys ansorgei*, 1 out of 65* Myonycteris torquata*, 1* Kerivoula *cf.* cuprosa*, and 1 out of 3* Phataginus tricuspis*.*

A *total of 162 bats, initially identified morphologically as* Epomops franqueti*, yielded inconclusive BLAST results based on high-quality partial or complete cytb gene sequences. BLAST analysis of these sequences resulted in* *top hits showing <93% pairwise nucleotide identity to unrelated taxa such as* Myonycteris *spp. or* Megaloglossus woermanni*. Phylogenetic analysis further revealed that these sequences clustered outside the* Epomops *genus, suggesting either misidentification, introgression, or the presence of nuclear mitochondrial DNA segments (NUMTs). While NUMTs are typically identifiable by premature stop codons or frameshifts, many of these divergent sequences were intact and fully translatable, raising the possibility that recent NUMT insertions or primer-driven preferential amplification of nuclear copies may underlie the observed patterns. Notably, in one case, a mother–pup pair yielded different cytb sequences: the mother’s matched* E. franqueti *with high confidence, while the pup’s was inconclusive, further supporting the presence of conflicting cytb targets. Additionally, on a subset of two individuals with suspected NUMTs, we amplified cytb using a different primer pair as part of a multiplex MinION assay (alongside 16S and COX1), and all three markers, including cytb, returned high-identity matches to* E. franqueti*: 97–98% nucleotide identity with* E. franqueti *for cytb, 93–97% for COX1, and 96.6–97.5% for 16S over fragments of 305 to 918 bp. This highlights that primer choice can critically influence amplification success and specificity, likely due to co-amplification of NUMTs. The occurrence of NUMTs has been documented in multiple bat taxa, but remains poorly studied and underreported, despite its known impact on mitochondrial-based inference* (37,38)*. This discrepancy will be explored further in future studies. For the purposes of the current study, we therefore retain the morphological identification of these individuals as* Epomops franqueti*.*

*Animal Serology*

The assay incorporated recombinant orthoebolavirus proteins, including glycoprotein (GP), nucleoprotein (NP) and viral protein 40 (VP40) from 5 different orthoebolaviruses: EBOV (NP, amino acids [aa] 488 to 739, variant Mayinga 1976; VP40, aa 31 to 326, variant Kissidougou-Makona 2014; GP-K, aa 1 to 650, variant Kissidougou-Makona 2014; GP-M, aa 1 to 650, variant Mayinga 1976); SUDV (NP, aa 361-738, variant Gulu; GP, aa 1 to 637, variant Uganda 2000; and VP40, aa 31 to 326, variant Gulu); BDBV (GP, aa 1 to 501, variant Uganda 2007; VP40, aa 31 to 326, variant Uganda 2007); RESTV (GP, aa 1 to 650) and BOMV (GP, aa 1 to 672). The GPs were produced in baculovirus-insect cell systems, except Bombali GP, which was expressed in HEK-293 cells. The NP and VP40 antigens were expressed in E. coli. Following sample dilution (corresponding to a final plasma dilution of 1/2000), we incubated 100 µL with 50 µL of magnetic beads coated with recombinant protein (2 µg protein/1.25 x 10^6^ beads) and washed hereafter. For bats, we added 0.1 µg/mL of goat anti-bat biotin-labeled IgG (Euromedex, Souffelweyersheim, France) to each well and incubated for 30 min at 400 rpm at room temperature. For rodents, we added biotin anti-mouse IgG (4 μg/mL)208 (Sigma-Aldrich B7022; Merck Life Science; Hoeilaart, Belgium) to each sample. Only animals for which blood samples were available were tested for the presence of orthoebolavirus antibodies. Shrews and elephant shrews were not analysed due to the unavailability of suitable secondary antibodies. After a new washing step, we added 50 µL of 4 µg/mL streptavidin-R-phycoerythrin (Fisher Scientific/ Life Technologies, Illkirch, France). We read the results using a BioPlex-200 (BioRad, Marnes-la-Coquette, France) or MagPix (Luminex, Austin, TX, USA), expressing them as median fluorescence intensity (MFI) per 50 beads.

***Social science study***

Study A:

To complement the ecological study, we aimed to identify the daily activities carried out by the inhabitants according to their socio-demographic differences (age, gender, education, social status within the village, economic activities) that can trigger a spillover and the spread of the disease within the social and ecological environment of a rural village. This study included a structured questionnaire related to animal capture, human behaviour and subsistence activities, and local people's awareness, perceptions, and knowledge of bats (e.g. the month of the year when bats are present in local forests, the presence of bat roosts or breeding sites, bat hunting techniques and conditions of meat consumption, and beliefs or practices (e.g. medical virtues associated with bats, knowledge of bat-borne diseases). We interviewed volunteers (both female and male, older than 18 years) living in Inkanamongo. We selected people from a wide range of occupations (i.e., traditional, professional, and self-employed), and with a variety of interests in the forest and wildlife to get a diversity of opinions. We stopped interviewing when new data did not procure new insights (data saturation). These interview sessions were combined with participant observations and informal discussions during the ecological team's research activities (such as collecting trapped animals, collecting specimens at the wild meat market), and on the activities and movements of residents within and between villages in the study area. Data from the questionnaire were collected on paper forms in Lingala by an anthropologist from the DRC, translated in French and encoded in an Excel table for statistical analysis using R software. Notes and audio recordings from participant observations and informal discussions were transcribed in a Word document. We conducted a thematic analysis focusing on the understanding of the interactions between the ecosystem and the inhabitants of the village of Inkanamongo.

Study B**:**

To select the six villages, we used a list made by the Tshuapa Provincial Division of Humanitarian Affairs and Solidarity, which was responsible for psychological support to the affected communities during and after the 2014 outbreak. The survey was conducted door-to-door; one person per household (female or male, older than 18 years) was invited to answer our questions. Data was collected among 117 persons, among whom 29 were women (24.8%) and 88 were men (75.2%). S3 Table shows the number of persons interviewed in every village. Data was collected in Lingala and/or Kimongo (locally spoken languages) and was simultaneously audio-recorded and registered manually through tablets in the REDCap software. A database (in an MS Excel file) was produced by REDCap. A team of research assistants based in Kinshasa translated the recordings into French. The French translations were automatically transcribed through the software SONIX. The information in the database was compared with the recorded, translated, and transcribed information. Relevant findings were contextualised through the participants' explanations (qualitative data) when possible.

In the six villages, we collected data about:

- Demographic characteristics of the respondent (age; gender; ethnicity; education level; religion; number of household members; social status (=role held in the community); primary activity; second and possible third occupation);
- Individuals’ interactions with the forest (reasons for and frequency of entering the forest; periods of highest frequency of entering the forest; animals spotted; people (from the same or another village) accompanying the respondent to the forest; wild meat selling, buying, consumption, and preparation for consumption; frequency of fetching water; people accompanying or dedicated to fetching water);
- Interactions between communities (individuals’ travel destinations, reasons for and frequency of travelling, reasons for spending the night outside their village) and within the community (mass gatherings and estimated attendance);
- Individuals’ health-seeking behaviours (under normal circumstances);
- Influence of rumours of an EVD outbreak on conducting daily activities.

***Maps***

Maps were made in QGIS v3.16.1 (39) using base layers imagery provided by EOX::Maps Sentinel-2 Cloudless (https://s2maps.eu). Shapefiles were obtained from the Common Geographic Reference Framework (ITOS, 2019) via Humanitarian Data Exchange (HDX; <https://data.humdata.org/dataset/cod-ab-cod>) and from Natural Earth (<https://www.naturalearthdata.com/downloads/>), a public domain dataset supported by the North American Cartographic Information Society (NACIS). Natural Earth is free for use in any type of project (<https://nacis.org/initiatives/natural-earth/>, <https://www.naturalearthdata.com/about/terms-of-use/>).

Continental, national and subnational administrative boundaries shapefiles were sourced from the Common Geographic Reference Framework (ITOS, 2019) via the Humanitarian Data Exchange (HDX): <https://data.humdata.org/dataset/cod-ab-cod>. These data are licensed under the Creative Commons Attribution for Intergovernmental Organisations (CC BY-IGO) (ITOS / HDX, 2019). Hydrological network was obtained from OpenStreetMap and OpenStreetMap Foundation, accessed via the Humanitarian Data Exchange (HDX), and is licensed under the Creative Commons Attribution International (CC BY) license (<https://data.humdata.org/dataset/hydrographie-lineaire-rdc-drc-water-courses>). Roads and paths were digitised as vector features using Sentinel-2 satellite images as a visual reference, resulting in an original geospatial dataset. Protected area boundaries (including national parks, World Heritage Sites, and reserves) in the Democratic Republic of the Congo were sourced from the World Database on Protected Areas (2016) and distributed by OpenAfrica (https://bulk.openafrica.net), under a Creative Commons Attribution 4.0 International license (CC BY 4.0).

**Supplementary results**

**Sociological study**

Interviews revealed widespread perceptions of environmental degradation and mixed understandings of zoonotic disease risk among the surveyed populations. In Study A (N=45), participants expressed strong awareness of environmental changes, with 65% perceiving a decrease in wildlife, 25% noting soil degradation and reduced crop production, and 32% observing a general decline in natural resources. These findings suggest a broad recognition of ecological stress and its impact on livelihoods.

Regarding behaviors related to Ebola, the data showed considerable about the disease and its transmission. In the bat-specific interview session (Study A; N=10), only one respondent believed that bats transmit diseases like Ebola, while five were unsure, and four believed bats do not pose such a risk. In Study B (N=103), when asked whether they would change their activities if they heard a rumor of Ebola in the area, 52.4% indicated they would modify their behavior in response, suggesting that perceived disease threats can influence actions even when knowledge is limited or uncertain.

**References supplementary material**

1. Report of an International Commission. Ebola haemorrhagic fever in Zaire, 1976. Bull World Health Organ. 1978;56(2):271–93.

2. Gryseels S, Mbala-Kingebeni P, Akonda I, Angoyo R, Ayouba A, Baelo P, et al. Role of wildlife in emergence of ebola virus in Kaigbono (Likati), Democratic Republic of the Congo, 2017. Emerg Infect Dis. 2020;26(9):2205–9.

3. De Nys HM, Mbala Kingebeni P, Keita AK, Butel C, Thaurignac G, Villabona-Arenas CJ, et al. Survey of Ebola viruses in frugivorous and insectivorous bats in Guinea, Cameroon, and the Democratic Republic of the Congo, 2015–2017. Emerg Infect Dis. 2018 Dec 1;24(12):2228–40.

4. Seifert SN, Fischer RJ, Kuisma E, Nkoua CB, Bounga G, Akongo MJ, et al. Zaire ebolavirus surveillance near the Bikoro region of the Democratic Republic of the Congo during the 2018 outbreak reveals presence of seropositive bats. PLoS Negl Trop Dis. 2022;16(6):1–11.

5. Leirs H, Mills JN, Krebs JW, Childs JE, Akaibe D, Woollen N, et al. Search for the Ebola Virus Reservoir in Kikwit, Democratic Republic of the Congo: Reflections on a Vertebrate Collection. J Infect Dis. 1999 Feb;179(s1):S155–63.

6. Lacroix A, Kingebeni PM, Kumugo SPN, Lempu G, Butel C, Serrano L, et al. Investigating the circulation of Ebola viruses in bats during the Ebola virus disease outbreaks in the Equateur and North Kivu provinces of the democratic republic of Congo from 2018. Pathogens. 2021;10(5).

7. Peeters M, Champagne M, Ndong Bass I, Goumou S, Ndimbo Kumugo SP, Lacroix A, et al. Extensive Survey and Analysis of Factors Associated with Presence of Antibodies to Orthoebolaviruses in Bats from West and Central Africa. Viruses. 2023;15(9).

8. Breman JG, Johnson KM, Van Der Groen G, Robbins CB, Szczeniowski M V., Ruti K, et al. A search for Ebola virus in animals in the Democratic Republic of the Congo and Cameroon: Ecologic, virologic, and serologic surveys, 1979-1980. J Infect Dis. 1999;179(SUPPL. 1):1979–80.

9. Hayman DTS, Yu M, Crameri G, Wang LF, Suu-Ire R, Wood JLN, et al. Ebola virus antibodies in fruit bats, Ghana, West Africa. Emerg Infect Dis. 2012;18(7):1207–9.

10. Olival KJ, Islam A, Yu M, Anthony SJ, Epstein JH, Khan SA, et al. Antibodies in Fruit Bats, Bangladesh. Emerg Infect Dis. 2013;19(2):270–3.

11. Pourrut X, Souris M, Towner JS, Rollin PE, Nichol ST, Gonzalez JP, et al. Large serological survey showing cocirculation of Ebola and Marburg viruses in Gabonese bat populations, and a high seroprevalence of both viruses in Rousettus aegyptiacus. BMC Infect Dis. 2009 Sep 28;9:159.

12. Pourrut X, Délicat A, Rollin PE, Ksiazek TG, Gonzalez JP, Leroy EM. Spatial and temporal patterns of Zaire ebolavirus antibody prevalence in the possible reservoir bat species. In: Journal of Infectious Diseases. 2007.

13. Ogawa H, Miyamoto H, Nakayama E, Yoshida R, Nakamura I, Sawa H, et al. Seroepidemiological prevalence of multiple species of filoviruses in fruit bats (Eidolon helvum) migrating in Africa. J Infect Dis. 2015 Oct;212(suppl 2):S101–8.

14. Hayman DTS, Emmerich P, Yu M, Wang LF, Suu-Ire R, Fooks AR, et al. Long-term survival of an urban fruit bat seropositive for ebola and lagos bat viruses. PLoS One. 2010;5(8):2008–10.

15. Leroy EM, Kumulungui B, Pourrut X, Rouquet P, Hassanin A, Yaba P, et al. Fruit bats as reservoirs of Ebola virus. Nature. 2005;438(7068):575–6.

16. Swanepoel R, Leman PA, Burt FJ, Zachariades NA, Braack LEO, Ksiazek TG, et al. Experimental inoculation of plants and animals with Ebola virus. Emerg Infect Dis. 1996 Dec;2(4):321–5.

17. Pawęska JT, Jansen van Vuren P, Kemp A, Storm N, Grobbelaar AA, Wiley MR, et al. Marburg virus infection in egyptian rousette bats, South Africa, 2013–2014. Emerg Infect Dis. 2018 Jun 1;24(6):1134–7.

18. Jones MEB, Schuh AJ, Amman BR, Sealy TK, Zaki SR, Nichol ST, et al. Experimental inoculation of egyptian rousette bats (Rousettus aegyptiacus) with viruses of the ebolavirus and marburgvirus genera. Viruses. 2015;7(7):3420–42.

19. Towner JS, Amman BR, Sealy TK, Reeder Carroll SA, Comer JA, Kemp A, et al. Isolation of genetically diverse Marburg viruses from Egyptian fruit bats. PLoS Pathog. 2009;5(7):e1000536.

20. Goldstein T, Anthony SJ, Gbakima A, Bird BH, Bangura J, Tremeau-Bravard A, et al. The discovery of Bombali virus adds further support for bats as hosts of ebolaviruses. Nat Microbiol. 2018 Oct;3(10):1084–9.

21. Forbes KM, Webala PW, Jääskeläinen AJ, Abdurahman S, Ogola J, Masika MM, et al. Bombali virus in mops condylurus bat, kenya. Emerg Infect Dis. 2019 May 1;25(5):955–7.

22. Karan LS, Makenov MT, Korneev MG, Sacko N, Boumbaly S, Yakovlev SA, et al. Bombali Virus in Mops condylurus Bats, Guinea. Emerg Infect Dis. 2019 Sep;25(9):955–7.

23. Lebarbenchon C, Goodman SM, Hoarau AOG, Minter G Le, Santos A Dos, Schoeman MC, et al. Bombali Ebolavirus in Mops condylurus Bats (Molossidae), Mozambique. Emerginf Infect Dis. 2022;28(12):2583–5.

24. Amman BR, Carroll SA, Reed ZD, Sealy TK, Balinandi S, Swanepoel R, et al. Seasonal pulses of Marburg virus circulation in juvenile Rousettus aegyptiacus bats coincide with periods of increased risk of human infection. Kawaoka Y, editor. PLoS Pathog. 2012 Oct 4;8(10):e1002877.

25. Bell JP, Tompkins AM, Bouka-Biona C, Sanda IS. A process-based investigation into the impact of the Congo basin deforestation on surface climate. J Geophys Res. 2015;120(12):5721–39.

26. Happold DCD, Happold M. Reproductive strategies of bats in Africa. J Zool. 1990;222(4):557–83.

27. Tuttle MD, Stevenson D. Time and synchrony of parturition. In: Kunz TH, editor. Ecology of Bats. New York: Plenum Press; 1982. p. 107–11.

28. Leroy EM, Epelboin A, Mondonge V, Pourrut X, Gonzalez JP, Muyembe-Tamfum JJ, et al. Human Ebola outbreak resulting from direct exposure to fruit bats in Luebo, Democratic Republic of Congo, 2007. Vector-Borne Zoonotic Dis. 2009;9(6):723–8.

29. Schmidt JP, Park AW, Kramer AM, Han BA, Alexander LW, Drake JM. Spatiotemporal fluctuations and triggers of Ebola virus spillover. Emerg Infect Dis. 2017;23(3):415–22.

30. Van Cakenberghe V, Gembu Tungaluna GC, Musaba Akawa P, Seamark E, Verheyen E. The bats of the Congo and of Rwanda and Burundi revisited (Mammalia: Chiroptera). Eur J Taxon. 2017;2017(382):1–327.

31. Monadjem A, Taylor PJ, Cotterill FP., Schoeman MC. Bats of Southern and Central Africa: A Biogeographic and Taxonomic Synthesis. African Zool. 2011;46(2).

32. Patterson BD, Webala PW. Keys to the Bats (Mammalia: Chiroptera) of East Africa. Fieldiana Life Earth Sci. 2012 Nov 29;6(6):1–60.

33. Kingdon J. The Kingdon Field Guide to African Mammals. 2nd ed. Lo. Bloomsbury; 2015. 640 p.

34. Makenov MT, Le LAT, Stukolova OA, Radyuk E V., Morozkin ES, Bui NTT, et al. Detection of Filoviruses in Bats in Vietnam. Viruses. 2023;15(9):1–9.

35. Amman BR, Bird BH, Bakarr IA, Bangura J, Schuh AJ, Johnny J, et al. Isolation of Angola-like Marburg virus from Egyptian rousette bats from West Africa. Nat Commun. 2020 Dec 1;11(1):1–9.

36. Jayaprakash AD, Ronk AJ, Prasad AN, Covington MF, Stein KR, Schwarz TM, et al. Marburg and Ebola Virus Infections Elicit a Complex, Muted Inflammatory State in Bats. Viruses. 2023;15(2):1–23.

37. Mao X, Dong J, Hua P, He G, Zhang S, Rossiter SJ. Heteroplasmy and ancient translocation of mitochondrial DNA to the nucleus in the Chinese horseshoe bat (Rhinolophus sinicus) complex. PLoS One. 2014;9(5):1–8.

38. Zhang G, Geng D, Guo Q, Liu W, Shufen LI, Gao W, et al. Genomic landscape of mitochondrial DNA insertions in 23 bat genomes: characteristics, loci, phylogeny, and polymorphism. Integr Zool. 2022;17(5):890–903.

39. QGIS Association. QGIS Geographic Information System.
