# Supplementary material for: Assessing Ebola virus circulation in the Tshuapa province (Democratic Republic of the Congo): A One Health investigation of wildlife and human interactions
Source: PLoS Pathog. 2025 Nov 26;21(11):e1013628. doi: 10.1371/journal.ppat.1013628 (PMC12680337; doi:10.1371/journal.ppat.1013628)
Supplement: S2 Supporting Information — (DOCX) [file ppat.1013628.s012.docx]

**S2 Supporting Information. Structured questionnaire of Study A – sub survey 1.**

**Questionnaire interview_ etude sociologique Inkanamongo**

Protocole EBOVAC3SUBECO698 V2.0

GROUP III : les dynamiques ebola-écosystème-moyens de subsistances.

Lieu et date :

Enquêteur/Enquêtrice :

Signature :

1. les informations personnelles de l’enquêté :……….

Sexe :……………………………………………….Age :…………..ans

Localité :………………………… ………………………………………………

Religion : ………………………………………………………………………….

Groupe social : ……………………………………………………………………

Tribu : ……………………………………………………………………………..

Niveau d’étude : …………………………………………………………………..

Propriété de terre (locataire, propriétaire, sans terre) ………………………………

Situation professionnelle (enseignant, cultivateur, notable, sans emploi, chasseur, pêcheur) : …………………………………………………………………………………………………...

Statut migratoire (autochtones, allochtones, citadins) : …………………………………………………………………………………………………

1. **Ressources animales**
2. **Quelles sont les animaux qui existent dans la région ?**

*(Afin de compléter la liste des animaux donnés d’abord spontanément, l’enquêteur parcourira la liste ci-dessous mais juste en donnant les noms d’animaux, sinon trop compliqué et trop long)*

1. **Les Primates:**
   1. Prosimiens (galagos),
   2. Simiens
      1. Cercopithécidés
      2. Hominidés.
2. **Les Chiroptères:**
   1. chauves-souris frugivores
   2. chauves-souris insectivores.
3. **Les Insectivores:** (hérissons, musaraignes)
4. **Les Lagomorphes:** lièvres.
5. **Les Rongeurs:** écureuils, anomalures, lièvres sauteurs, goundis, loirs, rats-taupes, rhizomyidés, porcs-épics, aulacodes, rongeurs spécialisés, gerbilles, souris et rats, muridés.
6. **Les Carnivores:** canidés, mustélidés, loutres, mangoustes, hyènes, genettes, civettes et nandinies, félins.
7. **Les Fourmiliers à écailles:** pangolins.
8. **Les Afrothériens:** oryctérope, taupes dorés, musaraignes-éléphants, potamogales, damans, éléphants.
9. **Les Périssodactyles:** équidés (zèbres, okapi), rhinocéros.
10. **Les Artiodactyles:** hippopotames, porcs sauvages, chevrotain aquatique, buffles, bovinés à cornes en spirale, céphalophes, antilopes naines et cobes, impala, alcélaphinés, antilopes chevalines, ovins et caprins.
11. **Quelle est la fréquence des animaux dans le milieu?** *(Est-ce qu’il/elle les rencontre souvent, pas souvent, quand en particulier,...)*
12. **Dix ans avant, est-ce que ce sont les mêmes animaux qui étaient les plus abondants?** oui ou non
    1. Si oui, lesquels?
    2. Si non, lesquels?
13. **Si il y a eu des changements, pourquoi ?**
14. le changement d'usage des sols (déforestation pour les besoins agricoles, perte des qualités du milieu naturel, etc.),
15. la surexploitation (chasse, etc.),
16. les espèces invasives et les maladies, la pollution,
17. le changement climatique.
18. **Quelles sont les techniques de chasse que vous utilisez ?**
19. Arme à feu
20. piège
21. flèche, lance
22. Filet
23. Chasse aux chiens
24. Autres:......
25. **Quelle est la bonne période de chasse ?**
26. Pluie
27. Sèche
28. **Pour quels animaux chassez-vous seul? Et pourquoi?**
29. **Pour quels animaux chassez-vous en groupe? Et pourquoi?**
30. **Acceptez-vous de chasser avec des personnes d’autres villages?**
31. **A partir de quel âge les enfants sont autorisés à chasser?**
32. **Comment chassent les enfants?**
33. **A quelle distance du village se fait la chasse ?**
34. 1 jour de marche
35. 2-3 jours de marche
36. Autres:......
37. **Quelle est la destination des produits de chasse?**
38. Consommation locale
39. Boende
40. Mbandaka
41. Bumba
42. Kinshasa
43. Autres:......
44. **En cas de vente, quel est le groupe d’animaux le plus vendu et pourquoi ?**
45. **Dans quel état vendez-vous le gibier ?**
46. Frais
47. Boucané
48. **Combien de fois chassez-vous dans un mois ?**
49. Une fois
50. Deux fois
51. Occasionnel
52. Autres:.....
53. **Combien de jours durent une session de chasse ?**
54. 1 jour
55. 2-5 jours
56. 6-9 jours
57. 10-15 jours
58. Autres:..........
59. **Par session(s) de chasse** *(voir réponse ci-dessus)***, vous ramenez combien de gibiers ? Et de quel groupe ?**
60. **Existent-ils les espèces animales interdites de la chasse dans votre milieu ?** Oui, Non
61. **Si oui, le(s)quel(s) ?**
62. **Existe- t-il un système de contrôle de la chasse dans votre milieu ?** Oui, Non
63. **Si oui, qui contrôle la chasse ?**

1. Environnement
2. Agence nationale de renseignement
3. Secteur 
4. Autres:....

**II. Pêche**

1. **Quels sont les poissons qui existent dans le milieu ?**
2. **Quels sont les groupes de poissons les plus abondants ?**
3. **Quelles sont les techniques de pêche que vous utilisées ?**

1. Hameçon 2. Filet 3. Écopage 4. Plantes Ichtyotoxique 5. Autres………………….

1. **Existe-t-il un système de gestion des poissons dans votre milieu ?** Oui, Non
2. **Si oui, lequel ?............................................................................................**
3. **Dix ans avant, est-ce que ce sont les mêmes poissons qui étaient les plus abondants ou il y a des changements ?** oui, non
4. **Si oui, pourquoi ?**1. Surpêche
   2. Utilisation des plantes Ichtyotoxique
   3. Pêche fantôme (utilisation des moustiquaires)
   4. Autres:.....
5. **Quelles catégories de personnes pratiquent la pêche dans votre milieu ?**
6. Hommes  2. Femmes 3. Jeunes filles 4. Jeunes garçons 5. Enfants
7. **A quel âge peut-on commencer à pêcher?**
8. **Quelle est la bonne période de pêche ?**

1. Etiage *(=Niveau moyen le plus bas d'un cours d'eau)*

2. Crue *(=Élévation du niveau dans un cours d'eau, un lac)*

**k) Existe-t-il des espèces des poissons interdites dans votre milieu ?** Oui, Non

l) **Si oui lesquelles ? Et pourquoi ?**........................................................................

**m) Quelle est la destination des produits de pêche ?**

1. Consommation locale
2. Boende
3. Mbandaka
4. Bumba
5. Kinshasa
6. Autres:.................

**n) En cas de vente quel est le groupe le plus vendu? Et pourquoi ?**

**o) Quelle est l’unité de vente ?** 1. Bassin 2. Tas 3. Autres mesures…………….

**p) Combien coûte une unité ?** …………………………………………………...

**q) Sous quelle forme vendez-vous les poissons ?** 1. Fumé 2. Frais

**r) Combien de fois vous pêchez dans une semaine ?** ……………………………

**s) Combien de jours durent une session de pêche ?** 1. 1 jour

2. 2-5 jours

3. 6-9 jours

4. 10-15 jours

5. Autres:.......

**t) Quelle quantité des poissons ramenez-vous ?**

……………………………………………………………………………………………………………………………………………………………………………………………………

**III. Ressources végétales**

- 1. Bois énergie

1. **Avec quoi vous préparez vos nourritures ?**1. Braise
   2. Bois mort

3. Autre:..........

1. **En cas de bois de chauffage, comment l’obtenez-vous** (ramassage de bois mort, abattus culturaux *(brûlis de cultures en particulier)*, coupe et séchage) ?
2. **Vendez-vous les bois de chauffage ?** Oui, Non
3. **A qui ?**1. Communauté locale
   2. Citadin
   3. Négociant

4. Autre:.........

1. **Comment vous rendez-vous à ces endroits?**
2. **Faites-vous des arrêts sur le chemin? Si oui, lesquels?**
3. **Quel est l’unité de mesure ?**1. Tas
   2. Stère
4. **Combien d’unités vendez-vous dans un mois ?**………………………………………………………………………………………
5. **Combien coûte une unité de bois de chauffe ?**…………………………………………………………………………………………..
6. **Quelles sont les espèces de bois de chauffe les plus préférées ? Et pourquoi ?**……………………………………………………………………………………..
7. **Pensez-vous que les bois utilisez disparaitront un jour ?** Oui, Non
8. **Si oui, comment pensez-vous régénérer la forêt ?**…………………………………………………………………………………………
9. **En cas de Makala, comment vous l’obtenez ?**1. Achat
   2. Fabrication
10. **Vendez-vous le Makala ?** Oui, Non
11. **Qui achète votre Makala ?**1. Communauté locale
    2. Citadin
    3. Négociant

4. Autre:........

1. **Quelles sont les espèces préférées? Et pourquoi ?**…………………………………………………………………………………………..
2. **Combien de fours faites-vous par mois? Et de quelle dimension ?**…………………………………………………………………………………….
3. **Quelle est l’unité de mesure ?**
   …………………………………………………………………………………………..
4. **Combien coute l’unité de mesure ?**
   …………………………………………………………………………………………..
   1. Bois d’œuvre
5. **Y a-t-il des scieurs de bois dans la région ?**

Oui, Non

1. **Quels sont les techniques qu’ils utilisent pour scier leurs bois ?**
2. Tronçonneuse
3. Scie de long
4. Autres:.......
5. **Quelles sont les espèces préférées ? et pourquoi ?**
6. **Quelle est la destination du bois ?**
7. Consommation locale
8. Boende
9. Mbandaka
10. Bumba
11. Kinshasa
12. Pays étranger
13. Hors continent
14. **Quelle est la quantité produite ?** *(A voir si il/elle calcule la quantité et comment)*
15. 1-5 m^3^
16. 6-15 m^3^
17. 16-30 m^3^
18. Autres
19. **Par mois vous abattez combien d’arbres ?**
20. **Avez-vous un permis d’exploitation ?** Oui, Non
21. **Si oui, où l’obtenez-vous ?**
22. **Quels sont les espèces d’arbres que vous préférez comme matériaux de construction?**
23. **Vendez-vous aussi les sticks ?** Oui, Non *(A voir si cela fait sens ici ou pas)*
24. **Quelle(s) est/sont leur destination ?**1. Consommation locale
    2. Boende
    3. Mbandaka
    4. Bumba
    5. Kinshasa
    6. Autres:............
25. **Comment vous rendez-vous à ces endroits?**
26. **Qui sont les acteurs impliquées ?**
27. Autochtones
28. Allochtones
29. Citadins

**IV. Produits forestiers non- ligneux (PFNL)** *(càd autre que le bois)*

1. **Quels sont les PFNL qu’on rencontre dans la région ?**
2. **Quel est usage de PFNL pour vous?**
3. Consommation
4. Usage traditionnel
5. Construction des habitations
6. Médicaments
7. Autres:...............
8. **Moyen d’obtention ?** Cueillette, autres:................
9. **Y en a-t-il qui sont vendus ?** Oui, Non
10. **Si oui, le(s)quel(s) ?**……………………………………………………………………………………………………………..
11. **Quelle est leur destination ?**
    1. Consommation locale
    2. Boende
    3. Mbandaka
    4. Bumba
    5. Kinshasa
    6. Autres:
12. **Quelle est la quantité vendue ?**………………………………………………………………………………………………….
13. **Quand est-ce que ces produits sont vendus ?**………………………………………………………………………………………
14. **Est-ce que l’utilisation des PFNL affecte l’état de la forêt** ?
    Oui, Non
15. **Unité de mesure et prix ?**……………………………………………………………………………………………
16. **Est-ce que les revenus obtenus améliorent le vécu des paysans ?**Oui, Non
17. **Dix ans avant, est-ce que ce sont les mêmes quantités de PFNL qui étaient obtenus ou il y a des changements ?** Oui, Non
18. **Si oui, quels sont ces changements éventuels ?**……………………………………………………………………………………………
19. **Agriculture**
20. **Quelles sont les principales cultures que vous pratiquez (en ordre d’importance)?**…………………………………………………………………………………………………..
21. **Dix ans avant, est-ce que ce sont les mêmes espèces qui étaient les plus exploitées?** Oui, Non
    1. **Si oui, lesquels?**
    2. **Si non, lesquels?**
22. **Si il y a eu des changements, quelles sont les raisons des changements éventuels ?**…………………………………………………………………………………………..
23. **Quelle est l’étendue moyenne que vous défrichez chaque année ?** *(A voir si il/elle mesure l’étendue et comment)*1. 0,50 ha
    2. 1 ha
    3. 2 ha
    4. Autres:.....
24. **Quelle est la répartition des cultures suivant la saisonnalité ?**……………………………………………………………………………………………….
25. **Quelle quantité pouvez-vous produire dans une saison ?**………………………………………………………………………………………………..
26. **Quelle est la destination de la production ?**1. Consommation locale
    2. Boende

3. Mbandaka
4. Bumba
5. Kinshasa
6. Autres:..........

1. **Comment vous rendez-vous à ces endroits?**
2. **Vous arrive-t-il de stocker votre production ?** Oui, Non
3. **Si oui, comment ?**
4. Tas circulaire
5. Grenier
6. Sac
7. Dans un récipient
8. Autre:...............
9. **Quels sont les problèmes que vous rencontrez dans la pratique de l’agriculture?**

1. Éloignement de champ
2. Perte de la forêt
3. Accidents naturels (destruction par des animaux, inondations,...)

4. Accidents provoqués par l’homme (vols, outils cassés, ...)

5. Autres:...................

1. **Connaissez-vous des problèmes éventuels de maladies ou d’insectes ravageurs des cultures ?** Oui, Non
   1. **Si oui, lesquels?**
2. **Quelles pourraient en être la/les cause(s) ?**1. Commerce ou autres mouvement migratoire
   2. Facteurs environnementaux : conditions météorologiques ou transports par le vent
   3. Insectes ou autres vecteurs pathogènes

4. Autres:...........

1. **Comment les résolvez-vous ?**………………………………………………………………………………………………
2. **Quelle est la durée de la jachère ?**1. 1-2 ans
   2. 3-5 ans
   3. Plus de cinq ans
   4. Autres:..........
3. **Par rapport à l’eau, quelles sont les sources utilisées pour l’agriculture?** (de la rivière, collecte de la pluie, puits, sources naturelles,..?)
4. **Comment acheminez-vous l’eau jusqu’à vos terrains?**
5. **Ecologie**
6. **Quel connaissez-vous de l’utilisation non durable de la biodiversité ?**

1. **Que représente pour vous vos terres et forêts** (valeur économique, culturelle, sociale et symbolique?)
   1. source de revenus
   2. importance vitale pour notre santé
   3. Les arbres et les forêts sont des régulateurs de climat
   4. Les forêts contribuent à notre bien-être
   5. Espaces sacrés
   6. Autres:......
2. **Comment vos parents ont-ils conservé les forêts et terres dans les temps ?**

……………………………………………………………………..

1. **Comment vous conservez les forêts et terres pour le moment ?**

…………………………………………………………………….

1. **Est-ce que vous avez l’impression que vos forêts et terres ont changée?**

…………………………………………………………………….

1. **Qu’est ce qui a changé ?**

……………………………………………………………………

1. **Comment est-ce qu’elles ont changé ?**

…………………………………………………………………...

1. **Comment se présente ce changement dans votre environnement et dans l’espace ?**

…………………………………………………………………..

1. **A quoi ce changement est dû selon vous ?**

…………………………………………………………………..

1. **Depuis quand observez-vous ce changement ?**

………………………………………………………………….

1. **Est-ce qu’au temps de vos ancêtres, grand parents et parents c’était la même chose ? et pourquoi c’est différent maintenant ?**

………………………………………………………………...

1. **Est-ce ces changements ont un effet pour votre village, pour vous, pour vos activités économiques ?**

………………………………………………………………….

1. **Comment vous vous êtes adaptés à ces changements ?**

…………………………………………………………………

1. **Est-ce que ces changements ont un effet sur vos cultures ?**

………………………………………………………………….

1. **Comment pensez-vous que vos forêts et terres vont évoluées ? A quoi ressembleront-elles dans 10 ans? Dans 20 ans?**…………………………………………………………………………………

**Complément au questionnaire d’enquête**

**1. En dehors de l’usage ordinaire que vous faites des ressources de la biodiversité, avez-vous des circonstances particulières lors desquelles vous recourez à certaines espèces animales spécifiques?**

Oui Non

**2. Si oui, lesquelles ?**

| **Circonstances** | **Cocher ici** |
| --- | --- |
| Intronisation du chef |  |
| Circoncision |  |
| Rituel religieux |  |
| Deuil |  |
| Dot |  |
| Naissance du bébé |  |
| Réconciliation |  |
| Accessoire dans la chasse et pêche |  |
| Porteuse de bonheur |  |
| Médecine |  |
| Ornement |  |
| Habillement |  |

**3. Quelles sont les espèces animales que vous utilisez dans ces circonstances ?**

| **Circonstances** | **Espèces utilisées** |
| --- | --- |
| Intronisation du chef |  |
| Circoncision |  |
| Rituel religieux |  |
| Deuil |  |
| Dote |  |
| Naissance du bébé |  |
| Réconciliation |  |
| Accessoire dans la chasse et pêche |  |
| Porteuse de bonheur |  |
| Médecine |  |
| Ornement |  |
| Habillement |  |

**4. En dehors de l’usage ordinaire que vous faites des ressources de la biodiversité, avez-vous des circonstances particulières lors desquelles vous recourez à certaines espèces végétales spécifiques?**

Oui Non

**5. Si oui, lesquelles ?**

| **Circonstances** | **Cocher ici** |
| --- | --- |
| Intronisation du chef |  |
| Circoncision |  |
| Rituel religieux |  |
| Deuil |  |
| Dote |  |
| Naissance du bébé |  |
| Réconciliation |  |
| Accessoire dans la chasse et pêche |  |
| Porteuse de bonheur |  |
| Médecine |  |
| Ornement |  |
| Habillement |  |

**6. Quelles sont les espèces végétales que vous utilisez dans ces circonstances ?**

| **Circonstances** | **Espèces utilisées** |
| --- | --- |
| Intronisation du chef |  |
| Circoncision |  |
| Rituel religieux |  |
| Deuil |  |
| Dote |  |
| Naissance du bébé |  |
| Réconciliation |  |
| Accessoire dans la chasse et pêche |  |
| Porteuse de bonheur |  |
| Médecine |  |
| Ornement |  |
| Habillement |  |

**7. Dans quelles activités journalières les enfants sont impliqués?**
