# Supplementary material for: Assessing Ebola virus circulation in the Tshuapa province (Democratic Republic of the Congo): A One Health investigation of wildlife and human interactions
Source: PLoS Pathog. 2025 Nov 26;21(11):e1013628. doi: 10.1371/journal.ppat.1013628 (PMC12680337; doi:10.1371/journal.ppat.1013628)
Supplement: S3 Supporting Information — (DOCX) [file ppat.1013628.s013.docx]

**S3 Supporting Information. Structured questionnaire of Study A – sub survey 2.**

**Human-bat contact and associated diseases**

**General context.** This is part of a series of interviews that have been conducted in the Democratic Republic of the Congo since 2017, in a number of Provinces. The main objective of these interviews is to implement a mechanism for regular and participatory monitoring of bats (i.e., ethnobiological surveys, bat population dynamics, and bat-borne disease ecology around bat colonies and breeding sites) in an environment where orthopoxviruses and filoviruses are thought to circulate periodically/seasonally in wildlife and humans. We targeted bats because their situation illustrates the complex relationships between emerging zoonotic diseases and their key role in this emergence (e.g. bats have been hunted for food and traditional medicine in many inhabited tropical forests).

**Specific context for these surveys.** This is the ethnobiological part of the research, which aims to assess local/indigenous people’s awareness, perception and knowledge of bats, the location and season/period/month of bat presence in the human-influenced forests, the presence of bat roost/breeding sites, bat hunting techniques, the conditions of bat meat for consumption, and beliefs/legends/practices (e.g. medical virtues associated with bats, knowledge of bat-borne diseases). We surveyed volunteers who agreed to be interviewed freely, knowledgeably and without paying. We interviewed people from a wide range of occupations (traditional, professional, and self-employed) and with a wide range of interests in forest and wildlife. We used a response-saturation approach, meaning that we stopped interviewing when new data didn’t add anything new to the understanding of the phenomenon, in order to gather a variety of information.

**Human-bat contact interview guide (French version)**

Répondant(e) (prénom + nom) ________________________________________________ Date ________ / _______ /___________

| N°  ____ | Province  ______________ | Territoire  _________________ | Secteur  _________________ | Localité  _____________________ |
| --- | --- | --- | --- | --- |

Waypoint du domicile de répondant : __ __ __ ___ ___ ___ __ __ __ __ __E ; __ __ __ __ __ __ __ __ __ S ; ____ ___ ___ ___ ___ m

**Données sociodémographiques du répondant**

Sexe : M  F  Année de naissance : _____________________________________

Tribu: ___________________________________________________________________

Activité principale: **AG CH PE CO AU:_____________________________**Diplôme le plus élevé obtenu :

AG : agriculture, CH : chasse, PE : pêche, CO : commerce, AU : autre

**Connaissance des chauves-souris et maladies associées**

Quel est le nom (ou terme) suivant dans votre/vos langues (préciser la langue au-dessus de la colonne) ?

| FRANÇAIS | LANGUE 1 _______________________ | LANGUE 2 _________________________ | LANGUE 3 _________________________ |
| --- | --- | --- | --- |
| Chauve-souris frugivore |  |  |  |
| Chauve-souris insectivore |  |  |  |

1. Qu’est-ce qu’une chauve-souris (aspect morphologique, comportement, alimentation, mode de vie) ? Définition ouverte, laisser la personne répondre et raconter.

**Morphologie**

**Comportement**

**Régime alimentaire**

**Lieux où elles habitent**

**Autres commentaires du repondant**

2. Savez-vous où il y a des groupes ou colonies de chauves-souris dans cette région ? OUI NONREFUS

Si oui, complétez le tableau suivant :

| Présence de colonies ou groupes de chauves-souris : | Habitat* | Nom du lieu/localité/forêt | N heures de marche | Nom du cours d’eau le plus proche de la colonie | Sont-elles là en ce moment ?** |
| --- | --- | --- | --- | --- | --- |
| Dans des troncs arbres |  |  |  |  | **O NI** |
| Sur des arbres (feuillage) |  |  |  |  | **O NI** |
| Dans des rochers/grottes |  |  |  |  | **O NI** |
| Sous un toit de maison |  |  |  |  | **O NI** |
| Autre 1 : ___________________ |  |  |  |  | **O NI** |
| Autre 2 : ___________________ |  |  |  |  | **O NI** |

*Habitat : (forêt, lieu-dit, village, champ, quartier, etc… ; **O=oui, N=non, I=ne sait pas

3. Si la présence de chauves-souris et de colonies est mentionnée, veuillez compléter le tableau ci-dessous pour chaque espèce en indiquant leur nom local et poser des questions sur la forme des colonies.

ESPECE 1 : Nom en langue locale : _____________________________________________________________________________

Nom latin ou français (à remplir par l’enquêteur) ________________________________________________________________

Cette colonie est-elle :  présente toute l’année ; par période ; ne sait pas ?

Veuillez cocher TOUS les mois où elles sont présentes :

| **J** | **F** | **M** | **A** | **M** | **J** | **J** | **A** | **S** | **O** | **N** | **D** |
| --- | --- | --- | --- | --- | --- | --- | --- | --- | --- | --- | --- |

**Consommez-vous ces chauves-souris ? O NREFUS**

Achetez-vous ces chauves-souris

vivantes ; prix d’achat pour un individu : ___________ FC (change 1$= _________________ FC)

mortes ; dans quel état :  boucané ;  frais ; prix d’achat : ___________FC

Vendez-vous ces chauves-souris

vivantes ; prix de vente pour un individu : ___________ FC

mortes ; dans quel état :  boucané  frais ; prix de vente : ___________FC

Chassez-vous vous-même ces chauves-souris ? O NREFUS

Si oui, par quel moyen ? (Décrire la technique de chasse, l’heure, les appâts utilisés, etc…)

4. Utilisez-vous des excréments de chauves-souris ? O NREFUS

Si oui, quelle utilisation en faites-vous ? (Décrire)

5. Y a-t-il des croyances ou légendes associées ou liées aux chauves-souris dans votre région ou famille ?

O NREFUS ; si OUI, lesquelles ? (Raconter, décrire - par exemple le tabou alimentaire, pourquoi ?)

6. Les chauves-souris ou des parties de chauves-souris ont-elles des vertus médicinales ?

OUINON NE SAIT PASREFUS ; si oui, compléter le tableau avec les usages par organe/partie.

| Organe | Vertu médicinale | Vertu magique | Vertu aphrodisiaque | autres |
| --- | --- | --- | --- | --- |
|  |  |  |  |  |
|  |  |  |  |  |
|  |  |  |  |  |

7. Pensez-vous que les chauves-souris transmettent des maladies ? O NREFUS

Si oui, lesquelles ? (Citer les maladies et donner le mode de transmission)

| Maladie | Nom en langue locale | Nom scientifique | Symptômes | Mode de transmission |
| --- | --- | --- | --- | --- |
| Maladie 1 |  |  |  |  |
| Maladie 2 |  |  |  |  |
| Maladie 3 |  |  |  |  |

8. Voulez-vous ajouter quelque chose ou avez-vous des histoires liées aux chauves-souris ?

Mande C. & Laudisoit A., 2017
