# Supplementary material for: Assessing Ebola virus circulation in the Tshuapa province (Democratic Republic of the Congo): A One Health investigation of wildlife and human interactions
Source: PLoS Pathog. 2025 Nov 26;21(11):e1013628. doi: 10.1371/journal.ppat.1013628 (PMC12680337; doi:10.1371/journal.ppat.1013628)
Supplement: S4 Supporting Information — (PDF) [file ppat.1013628.s014.pdf]

Demographics

Code ID	______________________________
2. Date:	______________________________
3. Site:	◯ Inkanamongo<br>◯ Ituku<br>◯ Bokalia<br>◯ Isaka<br>◯ Watsi Kengo<br>◯ Iyute<br>◯ Autre, Specifier ______
4. Genre:	◯ M<br>◯ F
5. Age	______________________________<br>(ans)
6. Origine:	◯ Autochtone<br>◯ Immigré/e: ______.<br>◯ Préfère ne pas répondre<br>((spécifier d'où))
7. Nombre de personnes vivant dans le foyer:	______________________________
8. Statut social:	☐ Chef de ménage<br>☐ Chef de clan<br>☐ Leader communautaire<br>☐ Membre de la communauté<br>☐ Autre (spécifier) ______
9. Groupe ethnique:	◯ Mongo<br>◯ Préfère ne pas répondre<br>◯ Autre, (spécifier) ______
10. Niveau d'éducation:	◯ Aucune éducation<br>◯ Ecole primaire non terminée<br>◯ Ecole primaire<br>◯ Ecole secondaire non terminée<br>◯ Ecole secondaire<br>◯ Université<br>◯ Préfère ne pas répondre<br>◯ Autre, (spécifier) ______

11. Religion:	◯ Catholique<br>◯ Protestante<br>◯ Armée du salut<br>◯ Message du temps de la fin (Branhamiste)<br>◯ FEPACO (Nzambe Malamu)<br>◯ Aucune religion<br>◯ Préfère ne pas répondre<br>◯ Autre, (spécifier) ______
12. Occupation principale:	◯ Chasseur<br>◯ Pêcheur<br>◯ Cultivateur/cultivatrice<br>◯ Eleveur/se<br>◯ Cueillette<br>◯ Enseignant/e<br>◯ Infirmier/e ou travailleur/se de santé<br>◯ Femme au foyer<br>◯ Etudiant/e<br>◯ Chômeur/se<br>◯ Autre (spécifier) ______

Completed by: ______ Date completed ______

Partie 1

2. PARTIE 1 : Interactions animaux-humains

13. Quelles sont les trois activités principales, listées par ordre de fréquence, que vous menez pour assurer des moyens de subsistance à votre famille ? (proposer : chasse, pêche, cueillette, agriculture, commerce, enseignement, autre)

(i) Activité 1: ______

Y a-t-il une période pendant laquelle vous pratiquez cette activité plus souvent que d'habitude ? ______
Si oui, specifier ______

Si oui, pourquoi pratiquez-vous cette activité plus souvent que d'habitude pendant cette période ? ______

(ii) Activité 2: ______

Y a-t-il une période pendant laquelle vous pratiquez cette activité plus souvent que d'habitude ? ______
Si oui, specifier ______

Si oui, pourquoi pratiquez-vous cette activité plus souvent que d'habitude pendant cette période ? ______

(iii) Activité 3: ______

Y a-t-il une période pendant laquelle vous pratiquez cette activité plus souvent que d'habitude ? ______
Si oui, specifier ______

Si oui, pourquoi pratiquez-vous cette activité plus souvent que d'habitude pendant cette période ? ______

16. Est-ce que la fréquence de cette activité change quand vous êtes au courant du risque d'une possible nouvelle épidémie d'Ebola (par exemple, quelqu'un a signalé des symptômes dans un village à coté) ?

◯ Oui, (spécifier)
◯ Non

17. Pourquoi ?

____

18. Comment est-ce que cette activité change quand vous êtes au courant du risque d'une possible nouvelle épidémie d'Ebola ?

____

Interactions avec la forêt : Trajectoires d'éléments humains, animaux et végétaux dans leur écosystème dans le Secteur de Djera et leurs variations

19. Vous rendez-vous dans la forêt parfois ?

◯ Oui
◯ Non

20. Si oui, pour quel objectif ?

- ☐ Chasser
- ☐ Pêcher
- ☐ Faire la cueillette
- ☐ Cultiver
- ☐ Puiser de l'eau
- ☐ Aller dans un autre village
- ☐ Autre, (spécifier) ______

21. Quels sont les animaux avec lesquels vous entrez le plus souvent en contact, dans la forêt ?

____
(list all)

22. Avec quelle fréquence vous rendez-vous dans la forêt ?

- ◯ Chaque jour
- ◯ Chaque deux jours
- ◯ Deux fois par semaine
- ◯ Une fois par semaine
- ◯ Deux ou trois fois par mois
- ◯ Une fois par mois
- ◯ Cela dépend de la saison
- ◯ Préfère ne pas répondre
- ◯ Autre, (spécifier) ______

23. Y a-t-il une période pendant laquelle vous vous rendez dans la forêt plus souvent que d'habitude ?

- ◯ Oui, (spécifier) ______
- ◯ Non

24. Si oui, pourquoi allez-vous dans la forêt plus souvent que d'habitude pendant cette période ?

____

25. Vous rendez-vous dans la foret moins souvent quand vous êtes au courant du risque d'une possible nouvelle épidémie d'Ebola (par exemple, quelqu'un a signalé des symptômes dans un village à coté) ?

- ◯ Oui
- ◯ Non

26. Si oui, pourquoi ?

____

27. Comment est-ce que cela affecte votre vie de tous les jours ?

____

Interactions avec d'autres espaces : Trajectoires d'éléments humains, animaux et végétaux de leur écosystème dans le Secteur de Djera vers d'autres localités et écosystèmes, en RDC ou ailleurs, et leurs variations

28. st-ce que vous quittez parfois votre village pour aller ailleurs ?

- ◯ Oui
- ◯ Non

29. Où allez-vous quand vous quittez votre village ?

____

30. Pour quel objectif?	☐ Visiter des membres de la famille<br>☐ Visiter des amis<br>☐ Se faire soigner<br>☐ Aller à l'école<br>☐ Vendre des produits<br>☐ Acheter des produits<br>☐ Autre, (spécifier) ______
31. Avec quelle fréquence quittez-vous votre village ?	◯ Chaque jour<br>◯ Chaque deux jours<br>◯ Deux fois par semaine<br>◯ Une fois par semaine<br>◯ Deux ou trois fois par mois<br>◯ Une fois par mois<br>◯ Cela dépend de la saison<br>◯ Préfère ne pas répondre<br>◯ Autre, (spécifier) ______
32. Avez-vous un autre endroit pour dormir en dehors de votre village, par exemple une propriété à côté de vos champs, là où vous pouvez passer la nuit ?	◯ Oui<br>◯ Non
33. Cela se trouve où ?	________________________________
34. Quand est-ce qu'il vous arrive de passer la nuit là-bas ?	____
35. Y a-t-il une période pendant laquelle vous quittez votre village plus souvent que d'habitude ?	◯ Oui, (spécifier) ______<br>◯ Non
36. Pourquoi quittez-vous votre village plus souvent que d'habitude pendant cette période ?	____
37. Quittez-vous votre village moins souvent quand vous êtes au courant du risque d'une possible nouvelle épidémie d'Ebola (par exemple, quelqu'un a signalé des symptômes dans un village à coté) ?	◯ Oui<br>◯ Non
38. Si oui, Pourquoi?	________________________________
39. Comment est-ce que cela affecte votre vie de tous les jours ?	____

Circonstances des interactions entre populations humaines, animales et végétales et leur variation

A1) Animaux : Accès à viande de brousse & poisson/chasse & pêche

40. Faites-vous la chasse et/ou la pêche ?
◯ Oui
◯ Non

41. Si oui, où allez-vous chasser la viande de brousse ou pêcher ?
____

42. Quand est-ce que vous chassez la viande de brousse ou pêchez, d'habitude ?
____

43. Quelle est la meilleure période pour aller chasser ou pêcher ?
____

44. Pourquoi ?
____

45. Quels animaux chassez ou pêchez-vous pendant cette période-là ?
____

46. Comment faites-vous la chasse de la viande de brousse ou la pêche ? Pourriez-vous nous décrire vos techniques et procédures de chasse ou pêche ?
____

47. Allez-vous chasser ou pêcher seul ou avec d'autres gens ?
◯ Seul
◯ Avec d'autres gens, (spécifier) ______

48. Allez-vous chasser ou pêcher avec des gens venant d'autres villages, parfois ?
◯ Oui, (spécifier) ______
◯ Non

49. Pourquoi allez-vous chasser ou pêcher avec des gens de ces villages-là ?
____

50. Y a-t-il une période spécifique pendant laquelle vous allez chasser ou pêcher avec des gens venant d'autres villages ?
◯ Oui, (spécifier) ______
◯ Non

51. Pourquoi allez-vous chasser ou pêcher avec des gens venant d'autres villages pendant cette période-là ?
____

52. Allez-vous chasser ou pêcher moins souvent quand vous êtes au courant quand vous êtes au courant du risque d'une possible nouvelle épidémie d'Ebola (par exemple, quelqu'un a signalé des symptômes dans un village à coté) ?
◯ Oui
◯ Non

53. Si oui, pourquoi ?
____

54. Comment est-ce que cela affecte votre vie de tous les jours ?

____

55. Y a-t-il une catégorie spécifique de gens dans ce village qui s'occupe de la chasse et/ou de la pêche ?

◯ Oui, ______ (spécifier ; si enfants: de quel âge ?)
◯ Non

56. Comment est-ce qu'ils sont perçus dans la communauté ? Comment est-ce que les gens interagissent avec eux ?

____

A2) Animaux : Vendre/consommer viande de brousse & poisson

57. Vendez-vous de la viande de brousse ou du poisson ?

◯ Oui
◯ Non

58. Où vendez-vous la viande de brousse ou le poisson ?

____

59. Consommez-vous de la viande de brousse ou du poisson ?

◯ Oui
◯ Non

60. Qui prépare la viande de brousse ou le poisson que vous consommez ?

☐ Moi-même
☐ Quelqu'un d'autre, (spécifier) {q64_prep_meatfish_who_specific}

Specifier

____

61. Comment est-ce qu'on prépare la viande de brousse ou le poisson ?

____

B) Végétaux : Cultures, fruits et autres espèces végétales

62. Cultivez-vous ou faites-vous la cueillette de fruits et autres espèces végétales ?

◯ Oui
◯ Non

63. Où est-ce que vous cultivez ou faites la cueillette de fruits et autres espèces végétales ?

____

64. D'habitude, quand est-ce que vous cultivez ou faites la cueillette de fruits et autres espèces végétales ?

____

65. Quand est la meilleure période pour faire la récolte de vos cultures ou la cueillette de fruits et autres espèces végétales ?

____

66. Pourquoi ?

____

67. Quels sont les cultures, fruits et autres espèces végétales que vous récoltez pendant cette période-là ?	__________________________________
68. Comment faites-vous la récolte de cultures, fruits et autres espèces végétales ? Pourriez-vous nous décrire vos techniques et procédures de récolte ?	__________________________________
69. Allez-vous récolter seul ou avec d'autres gens ?	◯ Seul<br>◯ Avec d'autres gens, (spécifier) ______
70. Allez-vous récolter avec des gens d'autres villages, parfois ?	◯ Oui, ______ (spécifier d'où)<br>◯ Non
71. Pourquoi allez-vous récolter avec des gens de ces villages-là ?	__________________________________
72. Y a-t-il une période spécifique pendant laquelle vous allez récolter avec des gens venant d'autres villages ?	◯ Oui, ______ (spécifier)<br>◯ Non
73. Si oui, pourquoi allez-vous récolter avec des gens venant d'autres villages pendant cette période-là ?	__________________________________
74. Allez-vous récolter moins souvent quand vous êtes au courant quand vous êtes au courant du risque d'une possible nouvelle épidémie d'Ebola (par exemple, quelqu'un a signalé des symptômes dans un village à coté) ?	◯ Oui<br>◯ Non
75. Si oui, pourquoi ?	__________________________________
76. Comment est-ce que cela affecte votre vie de tous les jours ?	__________________________________
77. Y a-t-il une catégorie spécifique de gens dans ce village qui s'occupe de la récolte ?	◯ Oui, ______ (spécifier ; si enfants: de quel âge ?)<br>◯ Non
78. Comment est-ce qu'ils sont perçus dans la communauté ? Comment est-ce que les gens interagissent avec eux ?	__________________________________

C) Eau

79. Allez-vous puiser de l'eau ?
◯ Oui
◯ Non

80. Où est-ce que vous allez puiser de l'eau?

____

81. Quand allez-vous habituellement puiser de l'eau ?

____

82. Quelle est la meilleure période pour aller allez puiser de l'eau ?

____

83. Pourquoi ?

____

84. Comment puisez-vous l'eau ? Pouvez-vous décrire vos techniques et procédures pour puiser l'eau ?

____

85. Allez-vous puiser de l'eau seul ou avec d'autres gens ?
◯ Seul
◯ Avec d'autres gens, ______ (spécifier)

86. Allez-vous puiser de l'eau avec des gens d'autres villages, parfois ?
◯ Oui, ______ (spécifier d'où)
◯ Non

87. Si oui, pourquoi allez-vous puiser de l'eau avec des gens de ces villages-là ?

____

88. Y a-t-il une période spécifique pendant laquelle vous allez puiser de l'eau avec des gens venant d'autres villages ?
◯ Oui, ______ (spécifier)
◯ Non

89. Si oui, pourquoi allez-vous puiser de l'eau avec des gens venant d'autres villages pendant cette période-là ?

____

90. Allez-vous puiser de l'eau moins souvent quand vous êtes au courant quand vous êtes au courant du risque d'une possible nouvelle épidémie d'Ebola (par exemple, quelqu'un a signalé des symptômes dans un village à coté) ?
◯ Oui
◯ Non

91. Si oui, pourquoi ?

____

92. Comment est-ce que cela affecte votre vie de tous les jours ?

____

93. Y a-t-il une catégorie spécifique de gens dans ce village qui s'occupe de puiser l'eau ?	◯ Oui, ______ (spécifier ; si enfants: de quel âge ?)<br>◯ Non
94. Comment est-ce qu'ils sont perçus dans la communauté ? Comment est-ce que les gens interagissent avec eux ?	________________________________
95.Où vous baignez-vous habituellement	________________________________
96. Est-ce que d'autres personnes s'y baignent aussi ? uniquement de votre village ou d'autres villages également ?	________________________________

Completed by: ______ Date completed ______

Partie 2

---

1. Pourriez-vous nous décrire votre journée habituelle. (si compliqué de répondre à cette question : demander de décrire la journée précédente et indiquer si on peut la considérer comme une journée habituelle)

____

---

2. Y a-t-il des journées qui sont différentes des autres ?

____

---

3. Qui sont les gens avec qui vous êtes habituellement en contact, dans votre village ?

____

---

4. Pendant quelles occasions participez-vous à des rassemblements, dans votre village ?

☐ Rencontres de famille
☐ Matchs de football
☐ Partager la boisson
☐ Discuter en groupe le soir
☐ Messe et rencontres religieuses
☐ Enterrements
☐ Autres cérémonies sociales (dotes, mariages, intronisations, etc.) : ______ (spécifier)
☐ Autre (spécifier) ______

---

5. Combien de gens participant à ces rassemblements, d'habitude ?

____

---

6. (demander pour chaque rassemblement :) Avec quelle fréquence participez-vous à ces rassemblements ?

◯ Chaque jour
◯ Chaque deux jours
◯ Deux fois par semaine
◯ Une fois par semaine
◯ Deux ou trois fois par mois
◯ Une fois par mois
◯ Cela dépend de la saison
◯ Préfère ne pas répondre
◯ Autre ______ (spécifier)

---

7. Avec combien de gens êtes-vous en contact, d'habitude, sur votre lieu de travail ?

____

---

8 (a). Entrez-vous en contact physique direct ou indirect lors de cette interaction?

____

---

8 (b). Achetez-vous la viande de brousse ou le poisson ?

◯ Oui
◯ Non

---

8 (c). Si oui, où achetez-vous la viande de brousse ou le poisson ?

____

8 (d). Quand achetez-vous la viande de brousse ou le poisson ?

◯ Plusieurs fois dans la semaine
◯ Une fois par semaine
◯ à 3 fois par mois
◯ Une fois par mois

9. Quand il vous arrive d'être malade et présenter des symptômes comme fièvre, diarrhée, vomissement, saignement, est-ce que la fréquence et la qualité des contacts avec ces gens changent ? (par exemple, vous les voyez moins souvent, vous prenez des mesures de précaution, ...)

____

10. Pourquoi?

____

11. Avec quelle fréquence montrez-vous un ou plusieurs de ces symptômes : fièvre, diarrhée, vomissement, saignement (symptômes)?

◯ Chaque jour
◯ Chaque deux jours
◯ Deux fois par semaine
◯ Une fois par semaine
◯ Deux ou trois fois par mois
◯ Une fois par mois
◯ Cela dépend de la saison
◯ Préfère ne pas répondre
◯ Autre ______ (spécifier)

12. Quand vous êtes malade et vous présentez des symptômes comme fièvre, diarrhée, vomissement, saignement, où allez-vous pour chercher un traitement ?

◯ A. Je vais à la pharmacie à ______ (spécifier le nom du village)
◯ B. Je vais au Centre de Santé/à l'hôpital à ______ (spécifier le nom du village)
◯ C. Je vais chez le médecin à ______ (spécifier le nom du village)
◯ D. Je vais chez un guérisseur traditionnel à ______ (spécifier le nom du village)
◯ E. Je vais chez un leader religieux (prêtre, pasteur, ...) à ______ (spécifier le nom du village)
◯ F. Je vais chez quelqu'un qui habite dans un autre village (voir Q14)
◯ G. Quelqu'un vient chez moi pour m'assister ou me soigner (voir Q16)

13. Pourquoi allez-vous chez cette personne/dans cet endroit ?

◯ Je fais confiance à cette personne/cet endroit
◯ Je suis satisfait du service fourni par cette personne/cet endroit ______ (expliquer)
◯ Cette personne/cet endroit se trouve près de chez moi
◯ Cette personne/cet endroit ne coute pas cher, le traitement est abordable
◯ Quelqu'un (qui?) m'a recommandé d'aller chez cette personne/cet endroit
◯ Autre, ______ (spécifier)

14. (si la réponse a Q12 est F) Si vous allez chez quelqu'un qui habite dans un autre village, qui est cette personne et où habite-t-elle ?

◯ Un membre de la famille (restreinte : qui ?) vivant à ______ (spécifier le nom du village)
◯ Un membre de la famille (élargie : qui ?) vivant à ______ (spécifier le nom du village)
◯ Un ami vivant à ______ (spécifier le nom du village)
◯ Un collègue vivant à ______ (spécifier le nom du village)
◯ Un/e infirmier/e vivant à ______ (spécifier le nom du village)
◯ Autre, {q14_who_outside_goother} (spécifier)

Autre

____

Contact physique direct avec le corps du malade

Autre

____

16. (si la réponse a Q12 est G) Si quelqu'un vient chez vous pour vous assister ou vous soigner, qui est cette personne et d'où vient-elle ?

◯ Un membre de la famille (restreinte : qui ?) vivant à ______ (spécifier le nom du village)
◯ Un membre de la famille (élargie : qui ?) vivant à ______ (spécifier le nom du village)
◯ Un ami vivant à ______ (spécifier le nom du village)
◯ Un collègue vivant à ______ (spécifier le nom du village)
◯ Un/e infirmier/e vivant à ______ (spécifier le nom du village)
◯ Autre, ______ (spécifier)

17. Pendant le traitement fourni par cette personne quand vous êtes malade, est-ce que cette personne entre en contact avec :

◯ Fluides corporels du malade (sang, vomissement, salive, urine, matières fécales, etc.)
◯ Habits, tissues, linges, assiettes, verres, etc. touchés par le malade
◯ Matériel médical (seringues, etc.) utilisé sur le malade
◯ Contact physique direct avec le corps du malade ______ (expliquer)
◯ Aucun contact entre malade et soignant
◯ Autre ______ (spécifier)

18. De manière générale, entrez-vous en contact physique direct avec des fluides corporels humains (urine/sueur/vomissement/sperme/autres fluides corporels) ?

◯ Oui
◯ Non

19. Si oui, d'où proviennent ces fluides?

20. Avec quelle fréquence êtes-vous en contact physique avec ces fluides ?

◯ Chaque jour
◯ Chaque deux jours
◯ Deux fois par semaine
◯ Une fois par semaine
◯ Deux ou trois fois par mois
◯ Une fois par mois
◯ Cela dépend de la saison
◯ Préfère ne pas répondre
◯ Autre ______ (spécifier)

21. (pour les travailleurs de santé) : portez-vous des EPIs quand vous êtes exposés à des fluides corporels humains ?

◯ Oui ______ (spécifier)
◯ No

22. De manière générale, entrez-vous en contact physique direct avec des fluides corporels animaux (urine/vomissement/autres fluides corporels) ?

◯ Oui
◯ No

23. Si oui, d'où proviennent ces fluides?

____

24. Avec quelle fréquence êtes-vous en contact physique avec ces fluides ?

◯ Chaque jour
◯ Chaque deux jours
◯ Deux fois par semaine
◯ Une fois par semaine
◯ Deux ou trois fois par mois
◯ Une fois par mois
◯ Cela dépend de la saison
◯ Préfère ne pas répondre
◯ Autre (q24_freq_animalfluid_other} (spécifier)

Specifier

____

Completed by: ______ Date completed ______
