# Supplementary figures and images for: Assessing Ebola virus circulation in the Tshuapa province (Democratic Republic of the Congo): A One Health investigation of wildlife and human interactions
Source: PLoS Pathog. 2025 Nov 26;21(11):e1013628. doi: 10.1371/journal.ppat.1013628 (PMC12680337; doi:10.1371/journal.ppat.1013628)

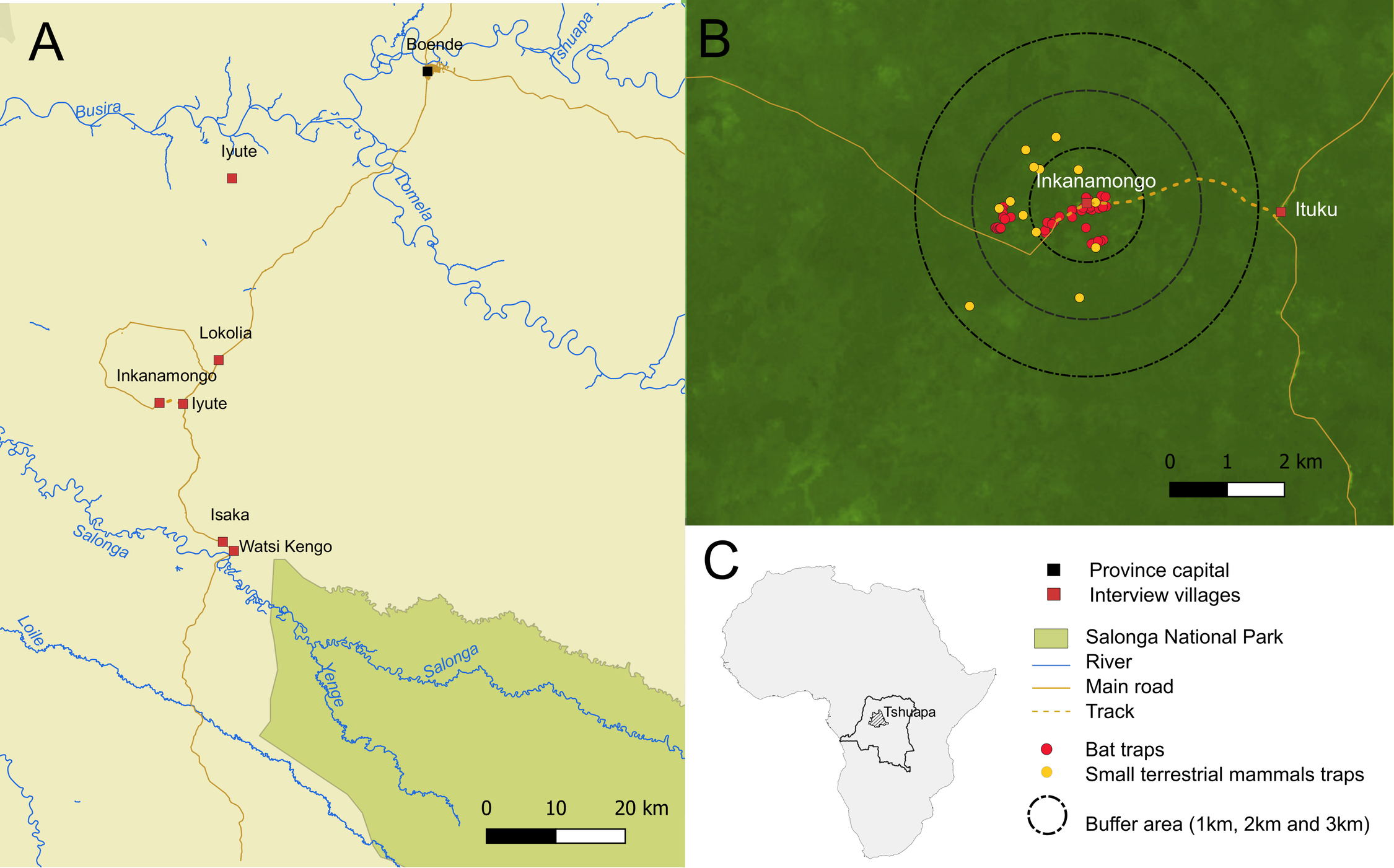

Supplement: S1 Fig — The interviewed villages are Inkanamongo, Ituku, Lokolia, Iyute, Isaka, and Watsi Kengo. Trapping took place in and around Inkanamongo. Base layers include: Sentinel-2 cloud-free satellite imagery from EOX::Maps Sentinel-2 Cloudless (EOX IT Services GmbH; https://s2maps.eu); administrative boundaries from the Common Geographic Reference Framework (ITOS, 2019) via HDX (https://data.humdata.org/dataset/cod-ab-cod) licensed under CC BY-IGO; physical features and country outlines from Natural Earth (public domain; https://www.naturalearthdata.com/downloads/); hydrological network from OpenStreetMap and OpenStreetMap Foundation via HDX (https://data.humdata.org/dataset/hydrographie-lineaire-rdc-drc-water-courses) under CC BY license; protected areas from the World Database on Protected Areas (2016) via OpenAfrica (https://bulk.openafrica.net) licensed under CC BY 4.0; roads and paths were digitised as vector features, resulting in an original geospatial dataset. All spatial data were used under open licenses and assembled using QGIS. (TIF) [file ppat.1013628.s015.tif]

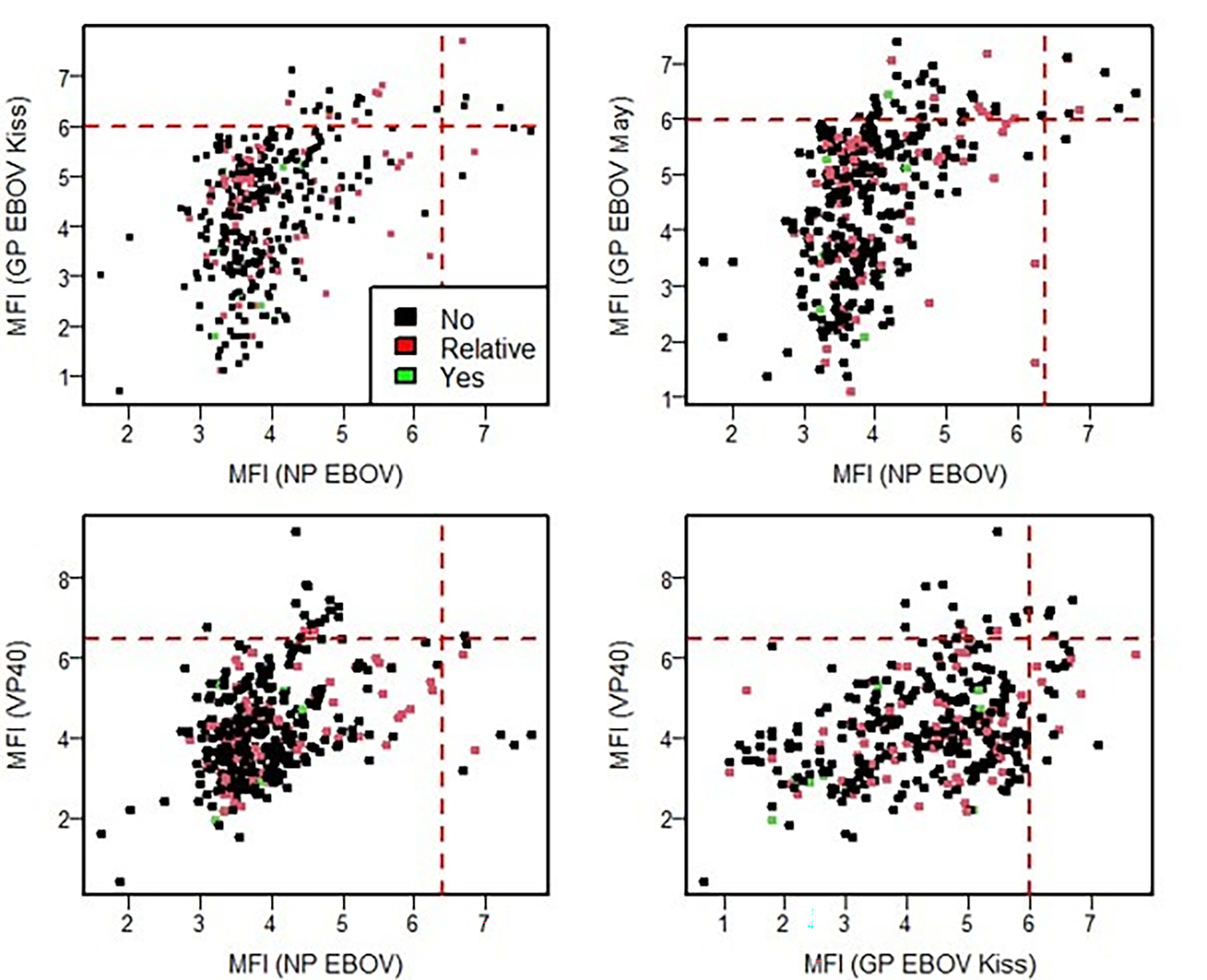

Supplement: S2 Fig — IgG antibody titres in inhabitants of Inkanamongo (DR Congo), expressed as the log of Median Fluorescent Intensity (MFI) values measured by Luminex for four Ebola virus antigens: nucleocapsid protein (NP), glycoprotein from the Kissidougou strain (GP-Kiss), glycoprotein from the Mayinga strain (GP-May), and viral protein 40 (VP40). The data are presented as follows: GP-Kiss and NP (top left), GP-May and NP (top right), VP40 and NP (bottom left), and VP40 and GP-Kiss (bottom right). Each dot represents an individual participant. Participants are classified by Ebola virus disease (EVD) exposure: no infection (black), infected relative (red), and participant became infected (green). The red dotted line represents the antigen-specific cutoff on the Luminex assay. (TIF) [file ppat.1013628.s016.tif]
