# Supplementary material for: Assessing Ebola virus circulation in the Tshuapa province (Democratic Republic of the Congo): A One Health investigation of wildlife and human interactions
Source: PLoS Pathog. 2025 Nov 26;21(11):e1013628. doi: 10.1371/journal.ppat.1013628 (PMC12680337; doi:10.1371/journal.ppat.1013628)
Supplement: Attachment — Submitted filename: Response to Reviewers.docx [file ppat.1013628.s018.docx]

Dear Editors and Reviewers,

We sincerely thank you for your thoughtful and constructive feedback on our manuscript titled “Assessing Ebola virus circulation in the Tshuapa province (Democratic Republic of the Congo): A One Health investigation of wildlife and human interactions (PPATHOGENS-D-25-00366). We appreciate the time and effort you have taken to evaluate our work.

We have carefully considered all comments and have revised the manuscript accordingly wherever possible. Below, we provide a detailed, point-by-point response to each reviewer’s comment. Our responses are coloured in blue. All changes made in the revised manuscript are clearly marked (underlined in this rebuttal, track changes in the revised manuscript) for your convenience. We have also addressed all technical/formatting issues mentioned in the ‘Journal requirements’ section of the decision email.

**Reviewers' Comments:**

 Reviewer's Responses to Questions

**Part I - Summary**
 Please use this section to discuss strengths/weaknesses of study, novelty/significance, general execution and scholarship.

Reviewer #1: van Vredendaal et al., Assessing Ebola virus circulation in the Tshuapa province (Democratic Republic of the Congo): A One Health investigation of wildlife and human interactions.

 These data reported show the collection of specimens from small mammals and inhabitants of a village at the site of an Ebola virus (EBOV) disease outbreak in 2014. EBOV viral RNA was not detected in any of the samples tested. In contrast, ortoebolavirus IgG antibodies were detected in bats, rodents and individual villagers. The manuscript is informative and reports original data.

Reviewer #2: The manuscript PPATHOGENS-D-25-00366 from van Vredendaal et al. is exploring the role of small mammals in EBOV ecology and especially the trace of possible transmission from potential wildlife (bats) or peridomestic (rodents) reservoirs to the human population in and around the Inkanamongo village (DRC) where an Ebola outbreak occurred in 2014. The approach is claiming to respect the « One Health » concept by combining molecular and serological studies in human and animals and by screening sociological and environmental factors via questionnaires. While no trace of EBOV RNA (orthoebolavirus) was detected in any samples, animal and human populations were seroreactive to orthoebolavirus suggesting a former (or current) circulation of “EBOV-like” between them. Anti-orthoebolavirus IgG antibodies were present in wildlife blood samples (4% bats and 14,2% rodents). Among the human participants, 120/380 individuals (31,5%) had IgG antibodies against at least 1 orthoebolavirus antigen and 12 (3%) against 2 antigens of the same orthoebolavirus.

 Although the molecular and serological analysis are seriously performed, trying to manage the inherent difficulties of the approach (absence of pos/neg controls...), the main weakness of the work consists in building a “One Health” chain of EBOV transmission between bats/rodents/human not sampled in the same year, nor in the same period of the year, sometimes in different locations

**Part II – Major Issues: Key Experiments Required for Acceptance**
 Please use this section to detail the key new experiments or modifications of existing experiments that should be absolutely required to validate study conclusions.

 Generally, there should be no more than 3 such required experiments or major modifications for a "Major Revision" recommendation. If more than 3 experiments are necessary to validate the study conclusions, then you are encouraged to recommend "Reject".

Reviewer #1: Ln 199. Has the multiplex serological Luminex-based serological assay been fully validated in line with international standards? Would you include the Specificity / Sensitivity values and reproducibility index in testing bat sera.

→ The multiplex serological Luminex-based assay used in our study follows an established methodology previously described in multiple studies (De Nys et al, 2018; Lacroix et al, 2021; Peeters et al, 2023). These prior investigations validated the assay for detecting orthoebolavirus antibodies in bats, demonstrating robust statistical validation, including cutoff determination and reproducibility testing.

Importantly, our study was conducted by the same laboratory that performed these previous investigations, ensuring methodological consistency and reproducibility. Specifically:

-We applied the same statistical approaches as used in Peeters et al. (2023), using a large dataset of 8741 wild bats to refine cutoff selection as described in the text.

-We followed negative control-based cutoff determination, using 145 negative samples from captive bats in European zoos, as done in De Nys et al. (2018) and Lacroix et al. (2021).

-Repeat testing was performed to confirm results, further ensuring assay reliability.

While this assay has not undergone formal validation against international regulatory standards (WOAH, FDA, EMA), the consistent methodology applied across multiple studies supports its robustness and suitability for serological analyses in bats.

Additionally, we do not have predefined sensitivity and specificity thresholds for bat sera testing. However, our results have been validated through a large panel of comparative samples, following the approach established in previous studies (Peeters et al., 2023; Lacroix et al., 2021).

By employing statistical methods applied to extensive datasets, including 8741 wild bats from Guinea, DRC, and Cameroon, we ensured a robust classification framework for identifying reactive samples. This methodology, based on multiple cutoff determination strategies, provides a strong alternative to predefined thresholds, ensuring reproducibility and consistency across sample sets.

Given the absence of standardized wildlife-specific reference samples, we believe this comparative approach offers the most robust validation strategy available for serological testing in bats.

De Nys HM, Mbala Kingebeni P, Keita AK, Butel C, Thaurignac G, Villabona-Arenas CJ, et al. Survey of Ebola viruses in frugivorous and insectivorous bats in Guinea, Cameroon, and the Democratic Republic of the Congo, 2015–2017. Emerg Infect Dis. 2018 Dec 1;24(12):2228–40. <https://doi:10.3201/eid2412.180740>

Lacroix A, Kingebeni PM, Kumugo SPN, Lempu G, Butel C, Serrano L, et al. Investigating the circulation of Ebola viruses in bats during the Ebola virus disease outbreaks in the Equateur and North Kivu provinces of the democratic republic of Congo from 2018. Pathogens. 2021;10(5). <https://doi.org/10.3390/pathogens10050557>

Peeters M, Champagne M, Ndong Bass I, Goumou S, Ndimbo Kumugo SP, Lacroix A, et al. Extensive Survey and Analysis of Factors Associated with Presence of Antibodies to Orthoebolaviruses in Bats from West and Central Africa. Viruses. 2023;15(9). <https://doi.org/10.3390/v15091927>

 Ln 285. Were any of the samples that tested positive confirmed by immunoblotting i.e. western blotting. If not, this should be undertaken.

→ At the outset of our study, we deliberately selected the Luminex assay platform over Western blotting based on several scientific and logistical considerations. Our study relied exclusively on dried blood spot (DBS) samples, which are inherently limited in both volume and protein concentration. Luminex technology is particularly well-suited for such sample types due to its high sensitivity and ability to detect multiple analytes simultaneously using minimal input material. Furthermore, Western blotting would have the same inherent problems as Luminex related to cutoff determination etc., as the same antigens would be used as the ones we coated on beads. Neutralization assays could partly solve this issue, but this is not possible on dried blood spots, and should be performed in BSL4 level conditions.

DBS samples offer significant advantages in terms of biosafety and logistics. The filter paper matrix inactivates infectious agents, allowing for safer handling, storage, and international transport, factors that are critical when working in remote or resource-limited settings.

The Luminex platform we used in our study has been thoroughly validated for the detection of EBOV antibodies in humans and bats. This assay showed a specificity and sensitivity similar to and higher than, respectively, commercial ELISAs when testing samples from 94 EBOV survivors and 108 patients from France (very likely true negatives). Considering samples positive for past EBOV infection only when they reacted with NP and GP proteins, the specificity was 99.1% (Ayouba et al., 2016). Given this high specificity and the multi-antigen approach of the Luminex assay, we consider confirmatory Western blotting unnecessary.

Given these factors, we determined that Luminex assays provided the most appropriate and informative approach for our study objectives and sample constraints.

Moreover, to ensure that our results are directly comparable with existing literature, we used the exact same Luminex-based methodology as described in De Nys et al. (2018), Lacroix et al. (2021), and Peeters et al. (2023).

Ayouba A, Touré A, Butel C, Keita AK, Binetruy F, Sow MS, et al. Development of a sensitive and specific serological assay based on Luminex technology for detection of antibodies to Zaire Ebola virus. J Clin Microbiol. 2016;55(1):165–76. <https://doi.org/10.1128/JCM.01979-16>

De Nys HM, Mbala Kingebeni P, Keita AK, Butel C, Thaurignac G, Villabona-Arenas CJ, et al. Survey of Ebola viruses in frugivorous and insectivorous bats in Guinea, Cameroon, and the Democratic Republic of the Congo, 2015–2017. Emerg Infect Dis. 2018 Dec 1;24(12):2228–40. <https://doi:10.3201/eid2412.180740>

Lacroix A, Kingebeni PM, Kumugo SPN, Lempu G, Butel C, Serrano L, et al. Investigating the circulation of Ebola viruses in bats during the Ebola virus disease outbreaks in the Equateur and North Kivu provinces of the democratic republic of Congo from 2018. Pathogens. 2021;10(5). <https://doi.org/10.3390/pathogens10050557>

Peeters M, Champagne M, Ndong Bass I, Goumou S, Ndimbo Kumugo SP, Lacroix A, et al. Extensive Survey and Analysis of Factors Associated with Presence of Antibodies to Orthoebolaviruses in Bats from West and Central Africa. Viruses. 2023;15(9). <https://doi.org/10.3390/v15091927>

Reviewer #2: As mentioned above, the main weakness of the work consists in building a “One Health” chain of EBOV transmission between bats/rodents/human not sampled in the same year, nor in the same period of the year, sometimes in different locations: indeed the animal samples were done in Inkanamongo village in May-June 2021 while the human samples were taken in December 2023. Concerning social interviews, a small part was done during the animal sampling in Inkanamongo village (May-June 2021) while the majority was performed in June 2022 in a larger Djerra sector (6 villages). Assuming the wildlife species seasonality, the current climate changes and possible landscape changes, as well as the probable versatility in human communities and occupation/behavior along the year, it is tedious to conclude holistically by comparing these different populations sampled/questioned at unrelated dates. Authors recognize themselves in lines 413-4 : « these discrepancies highlight the challenges of comparing seroprevalence data due to varying ecological settings, sampling dates, and seasons, all of which can influence the observed results ». Therefore :

→ We acknowledge that the asynchronous timing of data collection is unfortunate.

Our original intention was to conduct all components of the study concurrently. However, due to unforeseen logistical challenges, including the impact of the COVID-19 pandemic and the difficulty of accessing remote regions in Central Africa, synchronisation was not feasible. Despite these challenges, we believe the study retains its One Health value, as it integrates molecular and serological data from animals, serological data from humans, and sociological data that explore the interactions between humans, animals, and the environment. This is the first study in this region adopting such a comprehensive approach, making the study highly valuable.

Importantly, the phenomena under investigation are not highly time-sensitive. Serological markers, such as antibodies, persist for extended periods, often several years, providing reliable evidence of past exposure even when sampling is not perfectly aligned. Likewise, behavioural patterns captured through sociological assessments are unlikely to have changed significantly within the two-year window between the sampling and interviews.

As the reviewer notes, the animal sampling was done in a single village, where the outbreak occurred in 2014. Due to the high costs associated with fieldwork in such remote areas, the animal reservoir study could only be conducted in this village. The serological data come from the same village. However, sociological data were gathered from a broader region. We consider this a strength, as it provides valuable insights into human–animal interactions and potential exposure pathways at the regional level and in an area where this work has not been done before.

To our knowledge, this is the first study to apply a One Health approach in this region of Central Africa, combining ecological, epidemiological, and social science data. Despite the noted limitations, we believe our findings provide valuable insights and a foundation for future, more temporally harmonised investigations.

• It would be interesting to have more information about the bat seasonality (migration?) in this region as well as the climate characteristics and potential landscape modification from 2021 to 2023 seasons. Authors mention in lines 428-30 “For example, the seasonal arrival of E. helvum around Inkanamongo coincides with fruit ripening, increasing contact with humans through hunting... » but do not give precision about the corresponding months and how they are matching or not with the sampling.

→We agree that the seasonal ecology of Eidolon helvum is highly relevant to understanding patterns of human–bat contact and potential zoonotic risk. Eidolon helvum, the straw-colored fruit bat, is a widely distributed species and found across sub-Saharan Africa. Its movements are largely driven by the seasonal availability of fruiting trees, and it is known to form large, transient colonies in both urban and rural areas. Genetic studies have shown panmixia across the continent, indicating high levels of gene flow and long-distance movement, with no clear population structure even between distant regions (Peel et al., 2016).

In our study area (Inkanamongo, DRC), we observed the arrival of E. helvum between late May and June, coinciding with the local fruiting season. During the first two weeks of sampling in May 2021, no individuals of this species were captured. However, from the third week onward, E. helvum began appearing in our nets, and hunters were observed returning to the village with freshly killed individuals. Local participants also noted that the arrival of these bats signals the ripening of certain fruits, further supporting the link between bat movements and seasonal food availability.

As requested by the reviewer, we have now included this paragraph: lines 437-456 “Seasonal migration and foraging behaviours further complicate this dynamic, with the movements of animals potentially introducing the virus to new areas or intensifying local outbreaks (49). The seasonal migration of bats in large numbers has been hypothesised as another important risk factor in previous outbreaks (17). Ecological surveys conducted in Occidental Kasaï (DRC) suggested that the large seasonal migrations of fruit bats between April to May may have started the 2007 outbreak in Kasai. During this period, bats (including *H. monstrosus*, *E. franqueti*) were likely hunted in large numbers by villagers, corresponding to the observation that the putative index case obtained freshly killed bats from hunters. Even though no data can be found about the migratory behaviour of *H. monstrosus* and *E. franqueti*, hunters from these villages reported massive annual migrations of these species in the area (17). Similarly, during the Likati outbreak in 2017, a large seasonal colony of *E. helvum* bats was roosting at the Likati River from March to July (4). The human index case bought and consumed a large bat during this time. During our reservoir study in Inkanamongo, we witnessed the arrival of seasonally migrating *E. helvum* bats from May to June. Notably, while not a single specimen of this species was observed during the initial two weeks of our sampling in May 2021, we started to capture some in our nets in week three and noticed hunters returning to the village with them. Combined, these factors, age structure, reproductive cycles, and migration, create fluctuating ecological and epidemiological conditions that may influence EBOV transmission and seroprevalence, underscoring the need for targeted, seasonally informed surveillance strategies.”

Also, we acknowledge the reviewer’s suggestion to explore climate characteristics and potential landscape modifications between 2021 and 2023. While this is indeed a compelling direction for future research, such an analysis would require a dedicated study involving remote sensing, land-use change modeling, and ecological monitoring. Given the scope and objectives of the current manuscript, we have not included this level of environmental analysis, but we fully agree that it would provide valuable context for understanding bat ecology and zoonotic risk in the region.

Peel A, Baker K, Hayman D, et al. Bat trait, genetic and pathogen data from large-scale investigations of African fruit bats, *Eidolon helvum*. Sci Data. 2016;3:160049. <https://doi.org/10.1038/sdata.2016.49>

• Also, a complete “One Health” scope could explore, between bats/rodents and human, the potential role of livestock and domestic animals, particularly pigs, as intermediate hosts for Orthoebolavirus transmission. This is known for Reston virus and evidence of “Orthoebolavirus” seroprevalence in pigs has been shown in Sierra Leone, Guinea, Uganda and Ghana (doi.org/10.1093/infdis/jiy330 ; doi: 10.1111/tbed.13391; doi.org/10.1111/tbed.13822; doi.org/10.1292/jvms.22-0186 ; doi: 10.3201/eid3004.231034).

→ We fully agree that incorporating data from livestock is important to obtain a more comprehensive understanding of pathogen circulation across human, wildlife, and domestic animal interfaces. In response to this suggestion, we have now included livestock samples in our analysis. These samples were available to us and have now been tested using the same PCR methodology as was used for the other samples. All PCRs were negative, and these results have now been included in the revised manuscript in the Material and Method section (lines 197-199: “On top of that, we also sampled 34 livestock animals. We refer to the supporting information for more details on the sampling process (S1 Supporting Information).” and the numbers have been added in the results section lines 291-295 and Table 1: “We collected samples from 922 animals belonging to 60 different species within eight orders (Table 1). Details on the molecular species identification and screening results of all sampled specimens are provided in S4 Table. Overall, we did not detect any active EBOV infection in 1049 screened samples (464 pooled non-invasive samples and 585 pooled kidney and liver) from 919 individuals (Table 1).”.  Unfortunately, no blood samples were available and therefore we could not test seroprevalence in livestock.

The social part is another subject: in the “Results” it appears over-sized and -detailed compared to the molecular and serological parts that are more compacted; in the “Discussion” it resembles a review discussing evidence from previous literature rather positioning the data of the present article. This results in a manuscript looking an assemblage of two independent parts. Indeed :

 • most of the social observations are more descriptive than analysed in the context of the laboratory results. They could be shortened by integration in a table with only few words in the main text to highlight the important points.

→ We have now shortened this part by replacing it with a comparative table, lines 364-370:

“Two surveys in Inkanamongo and nearby villages revealed strong reliance on forest-based livelihoods and diverse wildlife use. **Table 3** summarises key findings on livelihood strategies, environmental perceptions, wildlife interactions, and health behaviours.

**Table 3. Key findings from sociological study.**

| **Theme** | **Key finding** | **Main figures** | **Study (N)** |
| --- | --- | --- | --- |
| Livelihood | - Multi-activity (hunting, fishing, farming, etc.) - Strong reliance on forest-based resources - Hunting mostly secondary or tertiary activity | 100% 🡪 agriculture  80% 🡪 hunting  80% had > 1 occupation | A (N=10)  bat-specific |
|  |  | 62.5% 🡪 agriculture  80% 🡪 hunting  70% 🡪 fishing  77.5% had >1 occupation | A (N=40) |
|  |  | 97% → agriculture  50% → hunting  57% → fishing  91.4% had >1 occupation | B (N=117) |
| Forest access | - Frequent interaction with forest environment | 46% → daily  32% → 2-3 times/ week | B (n=117) |
| Seasonality of resources use | - Seasonal variation in hunting and harvesting | 26/40 prefer wet season; 4/40 dry season; 10/40 no preference; bats used as fruit ripening indicators | A (N=40 +10)  General + bat-specific |
| Forest animal diversity | - Wide range of species observed in the forest | 13 taxonomic orders observed | A + B  (N=129) |
| Bushmeat and fish trade | - Local and regional trade, with dominance of local trade | 100% report local consumption; 42.5% mention trade to Boende | A (N=40) |
|  |  | 65% sell; 68.4% buy; mostly local trade; some trade with Boende | B (N=117) |
| Wild meat consumption | - Broad consumption | 68.4% buy wild meat; 93% consume wild meat | B (N=117) |
| Wild meat animal diversity | - Variety of species hunted and consumed - Partial confirmation of sociological data with molecular data | 9 orders hunted:  34.5% Cetartiodactyla (antelopes, sitatunga, bushpig)  29.8% Rodentia (porcupines, squirrels, rats)  28 species identified (molecular) | A + B  (N=129) |
| Cultural beliefs | - Ethno-cultural perceptions and symbolic meanings of bats - Cultural beliefs shape bat classification, hunting roles, and consumption taboos | Interviews with 10 adults from 6 Mongo clans (Ekolie, Djwangenda, Djiyoko, Nongo, Djeefo, Djosona); Ekolie hunt/sell but don’t consume bats; 3 Ekolie respondents reported that bat vocalizations signal misfortune | A (N=10)  bat-specific |

When combining data from the two surveys (A and B), Cetartiodactyla and Rodentia represented the orders most frequently reported as observed (Fig 2).”

• It would have been interesting to try to make a link between tested and interviewed individuals to see if their behaviour/occupation could be linked to sero-positivity. Unfortunately, testing (December 2023) was disconnected from the 40 interviews (May 2021). Even if some links could be recreated during testing, 2 years later, this would be only for a limited proportion of the 40 individuals, possibly too limited for statistics. For example is it possible to have more information about the activities, proximity to wildlife, of the 12/380 individuals found to be reactive against two antigens.

→ We appreciate the reviewer’s suggestion regarding the potential to link individual behaviors and occupations with seropositivity. We agree that such an analysis would offer valuable insights into risk factors associated with exposure. Unfortunately, we were unable to conduct our serological testing and interview datasets simultaneously, and no identifying information was preserved that would allow us to establish a direct match between individuals tested for serology and those interviewed about their interactions with wildlife.

• Line 482-end: this is a long paragraph to explain the confusion about the origin and the index cas in the district. Authors mention themselves the poor reliability of several answers from the population for different reason. How are they managing this potential bias?

→ We fully agree that the uncertainty surrounding the index case and the origin of the outbreak poses a significant challenge. We chose Inkanamongo as our field site based on the location identified by Maganga et al. (2014) as the outbreak’s point of origin. However, during fieldwork, we encountered conflicting local accounts regarding where the outbreak began. Our aim in including this paragraph was to explicitly acknowledge this ambiguity, which we see as reflective of the complex realities inherent in conducting field investigations during or after outbreaks.

Such uncertainty, possibly driven by recall bias, fear of stigma, or limited awareness in the early stages of the outbreak, may partly explain the ongoing difficulty in identifying the Ebola virus reservoir. By drawing attention to this issue, we hope to raise awareness of these challenges and help inform the design of future field studies facing similar circumstances.

According to local accounts, the alternative location suggested as the outbreak’s origin is only a few kilometers from Lokolia, which itself lies roughly 12 kilometers from our study site. While this spatial uncertainty may affect interpretations of virus circulation in rodents and sedentary bat species, it is important to note that many bat species can easily cover such distances during nightly foraging. Moreover, local hunters frequently undertake multi-day excursions that span these distances or more. For these reasons, we believe that our sampling site remains ecologically and epidemiologically appropriate for investigating virus circulation in relation to the outbreak.

Maganga GD, Kapetshi J, Berthet N, Kebela Ilunga B, Kabange F, Mbala Kingebeni P, et al. Ebola Virus Disease in the Democratic Republic of Congo. N Engl J Med. 2014 Nov 27;371(22):2083–91. <https://doi.org/10.1056/NEJMoa1411099>

**Part III – Minor Issues: Editorial and Data Presentation Modifications**
 Please use this section for editorial suggestions as well as relatively minor modifications of existing data that would enhance clarity.

Reviewer #1: Ln 85. I disagree with the statement that “detection of EBOV RNA proves infection at the time of sampling”. The presence of mRNA suggests an active infection. Viral RNA correlate with past infection. The text should be amended accordingly.

→ Thank you for this important correction. We have amended the text to reflect this distinction (lines 105-106). “Although the presence of antibodies indicates previous exposure to the virus and the detection of EBOV RNA suggests recent infection [11,15],”

Ln 89. A citation should be included after the sentence ‘Substantial evidence……’ One suggestion is Luis et al. A comparison of bats and rodents as reservoirs of zoonotic viruses: are bats special? Proc Biol Sci. 2013 Feb 1;280(1756):20122753. doi: 10.1098/rspb.2012.2753. PMID: 23378666; PMCID: PMC3574368.

→ Thank you for this suggestion. While Luis et al. (2013) provides a useful comparative perspective on bats and rodents as reservoirs of zoonotic viruses, it does not specifically address Ebola virus (EBOV). Moreover, the idea that bats are “special” reservoirs has been challenged in more recent literature, which emphasizes the role of taxonomic diversity and sampling effort. In our manuscript, the sentence in question serves as an introductory statement, and the paragraph that follows provides detailed support for this claim, including multiple references to earlier EBOV outbreaks, serological and molecular evidence, and comparisons with other filoviruses. Given this context, we believe the existing citations in the paragraph sufficiently support the statement and prefer not to add an additional reference at this point. We added the citations to this first sentence according to your comment and now it reads as follows (lines 110-111): “Substantial evidence suggests that bats, or at least particular species, may serve as a reservoir for EBOV (6,7,9,10,17–21).”

Ln 105: An additional reason is that many studies focus on catching and sampling healthy bats. This in proven to be unsatisfactory. The text should be amended accordingly.

→ We agree that relying solely on visual assessment to determine bat health status during field sampling is insufficient and potentially misleading.

Most pathogens known to infect bats do not produce visible clinical signs, even when infection is confirmed through molecular diagnostics or histopathological analysis. As a result, it is extremely difficult, if not impossible, to distinguish between “healthy” and “unhealthy” individuals in the field based on appearance alone. In many cases, bats appear outwardly healthy despite harboring pathogens, and signs of disease such as inflammation, tissue damage, or organ pathology are only detectable during necropsy or histological examination.

One might reasonably assume that there is an inherent sampling bias in field studies, particularly when bats are captured using mist nets or harp traps placed in forest corridors or at cave entrances. These methods tend to capture only those individuals that are actively flying, presumably the “healthier” portion of the population. In contrast, hand-netting within roosts may allow for the capture of less active or potentially unwell individuals. However, even this distinction may not fully address the issue, as experimental evidence shows that bats infected with high-consequence pathogens such as filoviruses often exhibit no outward signs of illness.

This is particularly evident in the case of filoviruses such as Marburg virus (MARV) and Ebola virus (EBOV). Experimental infections in Egyptian rousette bats (Rousettus aegyptiacus) have consistently shown that these animals can sustain systemic viral replication without exhibiting any clinical signs of disease. In the study by Paweska et al. (2016), bats inoculated with EBOV remained clinically well, maintained normal food intake, and showed no gross pathology or external injuries throughout the experiment. Similarly, Jones et al. (2015) reported that bats inoculated with five different ebolaviruses and MARV exhibited no clinical signs, behavioral changes, or mortality. More recently, Jayaprakash et al. (2023) confirmed that MARV and EBOV infections in R. aegyptiacus elicited a complex but muted inflammatory response, with no observable clinical symptoms despite detectable viral replication in tissues3.

The few exceptions where clinical signs are observable include rabies (lyssavirus) and White-Nose Syndrome (WNS), caused by Pseudogymnoascus destructans. These conditions can manifest in visible symptoms including White fungal growth on the nose, ears, wings, or tail, wing lesions and scarring, abnormal winter behavior, such as flying during daylight or in freezing temperatures, poor body condition due to fat depletion from frequent arousals during hibernation, paresis (weakness) and an inability to fly, hang properly, swallow properly or move about, seizures, tremors, unusual vocalisation or incessant licking, changes in behaviour, including agitation, aggression and approaching people).

Therefore, the absence of visible symptoms during capture in the investigation of filoviruses does not equate to the absence of infection. Most field studies, including ours, inevitably target bats that appear outwardly “healthy,” not due to sampling bias, but because clinical illness is rarely detectable in bats by visual inspection alone. This highlights the critical role of laboratory-based diagnostics in identifying subclinical or latent infections and the limitations of visual health assessments in field epidemiology.

Paweska, J. T., Storm, N., Grobbelaar, A. A., Markotter, W., Kemp, A., & Jansen van Vuren, P. (2016). Experimental Inoculation of Egyptian Fruit Bats (Rousettus aegyptiacus) with Ebola Virus. Viruses, 8(2), 29. https://doi.org/10.3390/v8020029

Jones, M. E. B., Schuh, A. J., Amman, B. R., Sealy, T. K., Zaki, S. R., Nichol, S. T., & Towner, J. S. (2015). Experimental Inoculation of Egyptian Rousette Bats (Rousettus aegyptiacus) with Viruses of the Ebolavirus and Marburgvirus Genera. Viruses, 7(7), 3420-3442. https://doi.org/10.3390/v7072779

Jayaprakash, A. D., Ronk, A. J., Prasad, A. N., Covington, M. F., Stein, K. R., Schwarz, T. M., Hekmaty, S., Fenton, K. A., Geisbert, T. W., Basler, C. F., Bukreyev, A., & Sachidanandam, R. (2023). Marburg and Ebola Virus Infections Elicit a Complex, Muted Inflammatory State in Bats. Viruses, 15(2), 350. https://doi.org/10.3390/v15020350

Ln 116. Additional topics also include collecting bat guano for use as fertilizer, religious practices (preying) in caves habituated by bats and hunting bushmeat.

→ We acknowledge that practices such as guano collection, religious rituals in bat-inhabited caves, and broader bushmeat hunting may also be relevant to zoonotic risk. However, we chose to focus our discussion on behaviors and socio-cultural factors that were most directly addressed in the studies cited and most relevant to our research context. We feel that a detailed discussion of these additional practices, while important, would go beyond the scope of this section.

Ln 244. Did the social science study include the following: medical and travel history.

 →The social science study did not include detailed medical or travel histories. Participants were asked about the frequency of specific symptoms but not about their broader medical history. Similarly, while respondents were asked whether they leave their village, including destinations and frequency, no detailed travel history was collected.

In the serology study, the questionnaire included whether participants had ever been diagnosed with Ebola Virus Disease (EVD) or experienced EVD-related symptoms. However, no additional medical or travel history was recorded. Furthermore, to maintain confidentiality, only anonymized study numbers were used for tested individuals, preventing direct identification of participants who were both tested and interviewed.

Ln 265. A structured questionnaire should be included in a supplementary file.

→ We have now added the structured questionnaires of study A and B as supporting information (S2 Supporting Information, S3 Supporting Information, S4 Supporting Information).

Ln 353. Does the consumption of wild meat by villagers include the consumption of bats? If so, the text should be amended accordingly.

→ As noted in the manuscript (lines 375–376), only one respondent in the bat-specific survey reported consuming bats. However, during the animal reservoir study, we observed hunters returning to the village with dead bats, indicating that bats may indeed be hunted and potentially consumed, despite limited direct acknowledgment. Interestingly, none of the interviewees from the general surveys (see Fig 2) reported hunting bats, which contrasts with our field observations.

As discussed in the manuscript (lines 493–504), this discrepancy between interview responses and field observations may reflect differing interests related to wildlife hunting, such as livelihoods or trade, or heightened awareness of health risks. This may lead individuals to be reluctant to admit to hunting or consuming bats, particularly following the 2014 Ebola outbreak. Although we did not directly observe bat consumption, these field observations suggest that such practices may occur but are likely underreported due to cultural or social sensitivities.

We believe this point is adequately addressed in the current manuscript but would be happy to clarify further if necessary.

Table 1 and Fig.1. provide supporting information and should be considered as Supplementary Files.

→ We have moved Fig 1 to the Supporting Information section and it is now labeled as S1 Fig. Consequently, the previous S1 Fig has been renumbered as S2 Fig.

However, we believe that Table 1 provides key information that is essential to include in the main manuscript. It offers a clear and immediate overview of the screened animal orders and the number of samples screened per order, which we feel is important for readers to grasp the scope of the study at a glance.

Additionally, detailed species-level data are already provided in the Supporting Information, as we felt that level of detail would be too extensive for the main text.

Reviewer #2: • Is pooling samples susceptible to render the test less sensitive (false negative)? Alternatively, is the nature of sampling possibly involved? Non-invasive samples in bats, not suitable organs in rodents. Marburg virus was isolated in bat spleen. Could this be discussed?

→ Thank you for this important comment. We agree that both pooling and the nature of sampling can influence detection sensitivity. In our study, samples from the same individual were pooled prior to extraction and testing. While pooling can reduce sensitivity, especially in cases of low viral load, no positive pools were detected, so deconvolution was not applicable. Prior studies used pooled organs as well, and were able to detect filovirus RNA in pools of up to four different organs (Kajihara et al., 2019; Makenov et al., 2023a).

Regarding tissue selection, we recognize that the spleen is a relevant organ for filovirus detection and has often been used in virus isolation efforts. We did collect spleen samples, however, due to resource constraints, we were unable to extract all available tissues from every individual and had to prioritize based on a broader screening strategy. Our choice of liver and kidney was based on both practical and scientific considerations (see below).

Although this manuscript focuses on Ebola virus, our field sampling was designed with broader future objectives in mind, including plans to screen for other viral taxa such as paramyxoviruses and hepaciviruses. Due to limited resources, we prioritized the collection and extraction of tissue types that are commonly suitable for detecting a wide range of viruses. Previous work has shown successful detection of filoviruses, including Marburg and Ebola viruses, from the liver and kidney, as well as isolation of filovirus from the liver, in addition to the spleen (Amman et al., 2012; Amman et al., 2015; Amman et al., 2020; Forbes et al., 2019; Jayaprakash et al., 2023; Kajihara et al., 2019; Makenov et al., 2023a; Makenov et al., 2023b). Therefore, while we acknowledge the relevance of the spleen as a target tissue, the selection of liver and kidney is also appropriate and well-supported by precedent.

We also recognize the value of non-invasive or minimally invasive sampling methods, such as oral and rectal swabs, which are commonly used in field surveillance. Although these sample types may yield lower viral loads and thus reduced sensitivity, they remain useful tools for broad surveillance, especially when full necropsy is not feasible. Previous studies have detected filovirus (Marburg virus, Ebola virus Reston, Ebola virus Zaïre, Lloviu virus, Bombali virus),  from oral and oropharyngeal swabs and feces in natural wild population or in experimental infection studies (Amman et al., 2015; Amman et al., 2020; Jayme et al., 2015; Lebarbenchon et al., 2022; Negredo et al., 2011; Riesle-Sbarbaro et al., 2024).

Amman BR, Carroll SA, Reed ZD, Sealy TK, Balinandi S, Swanepoel R, et al. (2012) Seasonal Pulses of Marburg Virus Circulation in Juvenile *Rousettus aegyptiacus* Bats Coincide with Periods of Increased Risk of Human Infection. PLoS Pathog 8(10): e1002877. <https://doi.org/10.1371/journal.ppat.1002877>

Amman BR, Jones ME, Sealy TK, Uebelhoer LS, Schuh AJ, Bird BH, et al. Oral shedding of Marburg virus in experimentally infected Egyptian fruit bats (Rousettus aegyptiacus). *Journal of wildlife diseases.* 2015;51(1):113-124. <https://doi.org/10.7589/2014-08-198>

Amman BR, Bird BH, Bakarr IA, et al. Isolation of Angola-like Marburg virus from Egyptian rousette bats from West Africa. *Nat Commun*. 2020;11(1):510. Published 2020 Jan 24. doi:10.1038/s41467-020-14327-8

Forbes KM, Webala PW, Jääskeläinen AJ, et al. Bombali Virus in Mops condylurus Bat, Kenya. Emerg Infect Dis. 2019;25(5):955-957. doi:10.3201/eid2505.181666

Jayaprakash AD, Ronk AJ, Prasad AN, Covington MF, Stein KR, Schwarz TM, Hekmaty S, Fenton KA, Geisbert TW, Basler CF, et al. Marburg and Ebola Virus Infections Elicit a Complex, Muted Inflammatory State in Bats. *Viruses*. 2023; 15(2):350. <https://doi.org/10.3390/v15020350>

Jayme SI, Field HE, de Jong C. *et al.* Molecular evidence of Ebola Reston virus infection in Philippine bats. *Virol J.* 2015;**12**;107. <https://doi.org/10.1186/s12985-015-0331-3>

Kajihara M, Hang’ombe BM, Changula K, et al. Marburgvirus in Egyptian Fruit Bats, Zambia. *Emerging Infectious Diseases*. 2019;25(8):1577-1580. doi:10.3201/eid2508.190268.

Lebarbenchon C, Goodman SM, Hoarau AOG, et al. Bombali Ebolavirus in Mops condylurus Bats (Molossidae), Mozambique. Emerg Infect Dis. 2022;28(12):2583-2585. doi:10.3201/eid2812.220853

Makenov MT, Le LAT, Stukolova OA, Radyuk EV, Morozkin ES, Bui NTT, Zhurenkova OB, Dao MN, Nguyen CV, Luong MT, et al. Detection of Filoviruses in Bats in Vietnam. *Viruses*. 2023a; 15(9):1785. https://doi.org/10.3390/v15091785

Makenov MT, Boumbaly S, Tolno FR, et al. Marburg virus in Egyptian Rousettus bats in Guinea: Investigation of Marburg virus outbreak origin in 2021. *PLoS Negl Trop Dis*. 2023b;17(4):e0011279. Published 2023 Apr 26. doi:10.1371/journal.pntd.0011279

Negredo A, Palacios G, Vázquez-Morón S, González F, Dopazo H, et al. (2011) Discovery of an Ebolavirus-Like Filovirus in Europe. PLOS Pathogens 7(10): e1002304. <https://doi.org/10.1371/journal.ppat.1002304>

Riesle-Sbarbaro, S.A., Wibbelt, G., Düx, A. et al. Selective replication and vertical transmission of Ebola virus in experimentally infected Angolan free-tailed bats. Nat Commun 15, 925 (2024). https://doi.org/10.1038/s41467-024-45231-0

• It will be important to precise in which recombinant system the proteins (GP, NP, VP40) have been produced since this may play a substantial role in the recognition by Abs (glycosylation, etc). For example, in lines 280-82, it is surprising to find bat cross-reacting with the GP or the VP40 proteins of different orthoebolaviruses and not with the NP which is globally less divergent between viruses. As well, lines 282-84 it is surprising that no bat recognise different antigens of the same orthoebolavirus. Would it be linked to the lower quantity/quality of one antigen? For example, the NP antigen seems slightly less reactive compared to VP40 and GP. Is it linked to its production or to its lower immunogenicity and more rapid decline of humoral immunity? Authors evoke this last controversial point in the literature in lines

→ We agree that the expression system used for recombinant protein production can significantly influence antigen quality, particularly in terms of folding and post-translational modifications, which directly impact antibody recognition. To clarify, the glycoproteins (GPs) used in this study were expressed in baculovirus insect cells systems, except for Bombali GP, which was produced in HEK-293 cells. Nucleoprotein (NP) and VP40 were both expressed in E. coli.

As the reviewer correctly points out, GPs from different orthoebolaviruses elicited cross-reactive antibody responses, a phenomenon that was also observed and addressed in the original paper that describes the development of the Luminex assay (Ayouba et al. 2016). This justifies the use of reactivity to two antigens for determining seropositivity. While we report animals reacting to a single antigen, we consider seropositivity more reliable only when there is reactivity to two different antigens from the same virus as mentioned in the material and methods (lines 231-233).

The observation that bats showed cross-reactivity to GP and VP40, but not to NP, despite NP being the more conserved protein, may be attributed to differences in immunogenicity, antibody persistence, or epitope accessibility. Since both NP and VP40 antigens were expressed in E. coli, differences in expression systems are unlikely to account for the lack of NP cross-reactivity. As the reviewer suggests, the weaker NP response may instead result from inherently lower immunogenicity or a faster decline of anti-NP antibodies following infection, as reported in studies such as Diallo et al. (2021). However, this pattern is not consistent across all studies. For example, Rimoin et al. (2018) reported that NP-specific antibodies could persist for decades post-infection, with similar numbers of individuals testing positive for NP and GP, and substantially more than for VP40. We agree this is an important consideration and have now added a statement in the revised manuscript to clarify this point and to acknowledge its potential impact on our results, see lines 404-411: “We observed lower reactivity to NP compared to GP and VP40 in bats, and no NP cross-reactivity. Since both NP and VP40 were produced in E. coli, this difference likely reflects antigen-specific immunogenicity or antibody kinetics rather than expression system. Some studies report faster waning of NP-specific antibodies (47), while others found NP responses persisting decades post-infection (48), suggesting variability across contexts. Our two-antigen criterion for positivity increases specificity but may miss past infections if one response, such as to NP, has declined. We therefore interpret single-antigen reactivity with caution.”

Ayouba A, Touré A, Butel C, Keita AK, Binetruy F, Sow MS, et al. Development of a sensitive and specific serological assay based on Luminex technology for detection of antibodies to Zaire Ebola virus. J Clin Microbiol. 2016;55(1):165–76. https://doi.org/10.1128/JCM.01979-16

460-3. The notion of the quality (production) of the antigen used in the test must be also discussed.

→ All antigens used in this study had a protein purity exceeding 90%, as determined by SDS-PAGE. As mentioned previously, the Luminex platform employed here has been thoroughly validated for the detection of EBOV antibodies in both humans and bats, demonstrating its robustness and suitability for serological screening (Ayouba et al., 2016). These validations support the quality and reliability of both the antigens and the assay system used in our study.

Ayouba A, Touré A, Butel C, Keita AK, Binetruy F, Sow MS, et al. Development of a sensitive and specific serological assay based on Luminex technology for detection of antibodies to Zaire Ebola virus. J Clin Microbiol. 2016;55(1):165–76. https://doi.org/10.1128/JCM.01979-16

• For rodents, authors mentioned “trapping in the villages”. Is it possible to precise if the traps were peri-domestic or in houses? The more seropositive rodent is Lophomorus rita (15/38). Interesting to observe that some L. rita samples can recognize different proteins of the same orthoebolavirus, even if from different species (EBOV and BDBV). Could Lophomorus rita be a candidate as filovirus reservoir compared to Mus or Rattus sp. Is it found in or around houses ? What are its preference in term of food, roosts, behaviour. During the interviews, did population mention or not the consummation of wild rodents?

→ In response, we explain in the supporting information that traps are placed in and around the houses. In the results, we now also mention that half of the rodents that showed reactivity against 1 or more antigens were captured in or around the houses (lines 334-335): “Half of the rodents that showed reactivity against at least one orthoebolavirus antigen were captured in or around the houses (19/38)”. Regarding the high seroprevalence observed in *Lophuromys rita* (15/38 individuals reacting to at least one orthoebolavirus antigen), we agree that this species warrants further investigation. While we are cautious to suggest that *L. rita* is a more likely filovirus reservoir than *Mus* or *Rattus* species, given the broader ecological and virological criteria required to support such a statement, the observed reactivity is notable compared to *Mus cf. gratus* (3/29 reacting with 1 or more antigens) and *Rattus rattus* (9/72 reacting to 1 or more antigens). However, our serological assay was not thoroughly validated for use in rodents, and as such, some of the observed reactivity could represent false-positive results. *L. rita* was captured across all sampled habitat types, with nearly half of the individuals (16/38) trapped in or around houses. Although species-specific ecological information on *L. rita* is limited, members of the *Lophuromys* genus are generally omnivorous, with a strong preference for invertebrates. During community interviews in Inkanamongo, residents did report the consumption of wild rodents, and mitochondrial *cytb* analysis confirmed *L. rita* as one of the species hunted. These findings further support the relevance of *L. rita* as a species of interest in future filovirus reservoir investigations.

• Among IgG reaction for at least two Orthoebolavirus, it is interesting to note the coupled EBOV/BDBV since BDBV was responsible for an outbreak In DRC in 2012 with fatal cases.

We thank the reviewer for this observation. Indeed, the coupled IgG reactivity to Ebola virus (EBOV) and Bundibugyo virus (BDBV) antigens in one participant is noteworthy, particularly considering the historical 2012 BDBV outbreak in the Democratic Republic of the Congo. This pattern of serological reactivity could either reflect cross-reactive antibody responses or potential past circulation of multiple orthoebolaviruses in the region. We have now included this point in the discussion (lines 473-476) and it reads as follow: “Notably, one participant was found to be seropositive for both EBOV and BDBV. Given that the only known outbreak of BDBV in the DRC happened over 800km from our study site, this observation rather suggests cross-reactivity between orthoebolaviruses.”

While EBOV and BDBV are distinct species within the *Orthoebolavirus* genus, some degree of serological cross-reactivity has been reported, particularly for IgG antibodies targeting the viral glycoprotein (GP) (King et al., 2019; Peeters et al., 2023). The participant in our study showed reactivity against the GP and VP-40 proteins of EBOV and BDBV (as well as the GP of Sudan virus). As cross-reactivity of VP40 proteins is less commonly reported, these results might imply cocirculation of EBOV and BDBV, though we cannot confirm this with our current data.

King LB, Milligan JC, West BR, Schendel SL, Ollmann Saphire E. Achieving cross-reactivity with pan-ebolavirus antibodies. *Curr Opin Virol*. 2019;34:140-148. https://doi:10.1016/j.coviro.2019.01.003

Peeters M, Champagne M, Ndong Bass I, Goumou S, Ndimbo Kumugo S-P, Lacroix A, Esteban A, Meta Djomsi D, Soumah AK, Mbala Kingebeni P, et al. Extensive Survey and Analysis of Factors Associated with Presence of Antibodies to Orthoebolaviruses in Bats from West and Central Africa. *Viruses*. 2023; 15(9):1927. https://doi.org/10.3390/v15091927

 Minor comment
 • Is it possible to decrease the intensity of green in fig 1 (lower square down right) to clearly distinguish the points indicating capture sites.

→ We have adapted the map and decreased the intensity of green. The map is now presented in S1 Fig.
