# Supplementary material for: Assessing Ebola virus circulation in the Tshuapa province (Democratic Republic of the Congo): A One Health investigation of wildlife and human interactions
Source: PLoS Pathog. 2025 Nov 26;21(11):e1013628. doi: 10.1371/journal.ppat.1013628 (PMC12680337; doi:10.1371/journal.ppat.1013628)
Supplement: Attachment — Submitted filename: Response_to_Reviewers_auresp_2.docx [file ppat.1013628.s019.docx]

Dear Editors and Reviewer,

Thank you once again for your continued evaluation of our manuscript entitled *“Assessing Ebola virus circulation in the Tshuapa province (Democratic Republic of the Congo): A One Health investigation of wildlife and human interactions”* (PPATHOGENS-D-25-00366)*.* We are grateful for the additional feedback, which has helped us to further clarify and strengthen the Methods section. In particular, we have expanded the description of the serological assay to highlight its validation, reproducibility, and rationale for use. Below, we provide our response to this point as well as the updated text that has been added to the manuscript.

**Additional Editor Comments:**

Editor:

Please summarise your response to Reviewer 1's question regarding the multiplex serological Luminex-based serological assay in a few sentences and add them to the methods section.

→ Part of our response to Reviewer 1’s question, including cutoff determination and use of negative controls, was already included in the *Methods* section. To further clarify and strengthen this part of the manuscript, we have now expanded the description to emphasize assay validation, reproducibility, and the rationale for selecting the Luminex platform, as well as its performance compared to commercial ELISAs (lines 246-253): “*This assay has been extensively validated in prior bat studies by our laboratory (6,9,10), demonstrating reproducibility and robust statistical validation. We selected the Luminex platform over Western blotting or neutralization assays because of its high sensitivity, low sample volume requirements, and compatibility with dried blood spots, which also offer major biosafety and logistical advantages. The same methodology has been applied for EBOV antibody detection in human sera where it showed specificity and sensitivity comparable to or higher than commercial ELISAs (44), supporting its broader applicability for serological analyses in wildlife.”*

**Reviewers' Comments:**

Reviewer's Responses to Questions

**Part I - Summary**
Please use this section to discuss strengths/weaknesses of study, novelty/significance, general execution and scholarship.

Reviewer #2: Authors have done a clear effort to precisely respond to the reviewer's comments. Most of the weaknesses outlined by the reviewers have been (1) frankly recognised when impossible to clear them or (2) responded and eventually amended in the manuscript wherever possible to do. In both cases, an extended discussion with relevant references shows that authors are expert in the topic.

The response concerning the patchworking of dates, seasons and places of sampling is based on the limitation of studies due to COVID and regional instability and the limited importance of matching human and animal sampling period for serology since antibodies persist in blood. The latter is possible on the one hand, but on the other hand the litterature is also scaterred with examples where the antibody level can stronlgy evolve in 2,5 years (May 2021, December 2023)...

A strong point of the work is the tentative to respect the One Health context by focusing a specific village (Inkanamongo) even if at different periods. Also, the text has benefited of a interesting and substancial addition the seasonal aspect with precise examples underlining a link between consumption of freshly roosting bats and infection.

→ We sincerely thank the reviewer for this constructive and encouraging evaluation of our revised manuscript. We are glad that the additions on the One Health perspective and seasonal aspects were considered valuable.

Regarding the comment on antibody persistence, we agree that antibody kinetics in humans are heterogeneous, with studies reporting up to 95% loss of detectable antibodies within 0.5–2 years (Adaken et al. 2021), while others show persistence in over 59-76% of individuals, depending on glycoprotein, after 5 years (Diallo et al. 2021) and in some cases even up to several decades (Rimoin et al. 2017). We note, however, that our study does not attempt a direct comparison between human and animal serology results. Rather, we present these datasets in parallel to provide complementary insights within the same ecological and social context. As such, while the variability of antibody persistence is an important limitation to acknowledge, it does not alter our main conclusions.

**Part II – Major Issues: Key Experiments Required for Acceptance**
Please use this section to detail the key new experiments or modifications of existing experiments that should be absolutely required to validate study conclusions.

Generally, there should be no more than 3 such required experiments or major modifications for a "Major Revision" recommendation. If more than 3 experiments are necessary to validate the study conclusions, then you are encouraged to recommend "Reject".

Reviewer #2: All questions in this part have been responded for the satisfaction of Reviewer n°2

**Part III – Minor Issues: Editorial and Data Presentation Modifications**
Please use this section for editorial suggestions as well as relatively minor modifications of existing data that would enhance clarity.

Reviewer #2: All questions in this part have been responded for the satisfaction of Reviewer n°2
